# CG4968 positively regulates the immune deficiency pathway by targeting Imd protein in *Drosophila*

**DOI:** 10.7717/peerj.14870

**Published:** 2023-02-07

**Authors:** Qingyang Li, Chao Zhang, Chuchu Zhang, Renjie Duan, Yongzhi Hua

**Affiliations:** School of Life Sciences, Anhui Agricultural University, Hefei, China

**Keywords:** CG4968, Dub, Imd, K48-linked ubiquitination, *Drosophila melanogaster*

## Abstract

*Drosophila melanogaster* relies solely on innate immunity to defend against various microbial pathogens. Although it is well-known that the adaptor protein Imd undergoes K63-linked ubiquitination to activate the downstream signaling cascades, its involvement with K48-linked ubiquitination and what is responsible for controlling this modification remain largely unknown. In this study, we explored the immunological function of *CG4968*, which encodes a typical ovarian tumour-associated protease (OTU)-type deubiquitinase (Dub) in flies. Our *in vitro* and *vivo* evidence demonstrated that CG4968 plays a positive role in governing the immune deficiency (IMD), but not the Toll innate immune response in an OTU domain-dependent manner. Mechanistically, we found that CG4968 is associated with Imd to restrict its K48-linked ubiquitination, thereby contributing to its turnover. Collectively, our study uncovered a novel regulatory mechanism involving the K48-linked ubiquitination of Imd in *Drosophila* innate immunity.

## Introduction

*Drosophila melanogaster* (fruit flies) have evolved a strong innate immune system to defend themselves against external bacterial infections when living in a complex and rich microbial environment. Unlike mammals, *Drosophila* do not have adaptive immunity, and they rely primarily on their strong innate immune system for defence (Arch et al., 2022; Fuse et al., 2022). This innate immune response includes both cellular and humoral immune defences. The humoral immune response mainly involves the Toll and the immune deficiency (IMD) pathways, which share great similarities with the mammalian MyD88-dependent Toll-like receptor (TLR) and tumor necrosis factor receptor pathways (TNFR), respectively (Anthoney, Foldi & Hidalgo, 2018; Prakash, Roychowdhury-Sinha & Goto, 2021). It has been suggested that fungi and most Gram-positive bacteria are targeted by the Toll immune pathway, whereas the IMD pathway targets some types of Gram-negative bacteria and Gram-positive bacteria as well (Maitra et al., 2019). In the Toll immune pathway, Spaetzle is activated by the hydrolysis of an aminoprotease cascade protein upon infection and binds to the transmembrane receptor Toll, leading to the phosphorylation and degradation of the IκB factor Cactus and the nuclear translocation of NF-κB family members Dorsal or Dif, which induce the expressions of downstream antimicrobial peptides (AMPs) such Drosomycin (Drs) and Metchnikowin (Mtk) (Fitzgerald & Kagan, 2020). In the IMD pathway, Imd can recruit the Tab2/Tak1 complex to bind after K63-linked ubiquitination. The activated IKK complex phosphorylates NF-kB family member Relish, which releases its N-terminal and transfers it into the nucleus to start the expression of AMPs, such as Attacin (Att) and Cecropin (Cec), by fat body cells, thus killing the pathogen (Russell et al., 2021).

Ubiquitin (Ub) is composed of 76 amino acids and widely exists in eukaryotic cells (Song & Luo, 2019). Ub can covalently bind to the intracellular protein and affect biological processes including transcription, DNA damage signaling, DNA repair, and cell cycle progression by regulating protein interactions and enzyme activity through ubiquitination (Liang et al., 2019; Puvar et al., 2020). Ubiquitination, an orderly and diverse modification of protein, is a multi-step enzymatic reaction mediated by Ub-activating enzymes, Ub-congregating enzymes, and Ub ligases (Deng et al., 2020). Ubiquitination can have a variety of covalent linkages. When a single Ub is attached to the substrate protein, it is called mono-ubiquitination. When more Ub are attached to a single Ub molecule through one of the internal seven lysines (K6, K11, K27, K29, K33, K48, or K63) to form a linear ubiquitin chain, that is called poly-ubiquitination (Wang et al., 2020). The diversity of Ub chains plays an important role in complex cellular processes, and K48 and K63-linked ubiquitination have been most widely studied. The K48-linked ubiquitin chain induces the degradation of modified protein through 26s proteasome, and this process determines the protein’s stability (Bai et al., 2020). The K63-linked Ub chain can form a scaffold to recruit downstream protein binding and trigger the cascade effect of signal transduction (Cockram et al., 2021).

The Ubiquitination of a protein is reversible, and deubiquitinase (Dub) mainly causes the deubiquitination of proteins (Deng et al., 2019). Dubs specifically hydrolyze Ub molecules in proteins or precursor proteins bind to ubiquitinated substrates and hydrolyze the ester, peptide, or isopeptide bonds at the carboxyl terminus of Ub, modulating the levels of protein ubiquitination in cells to control a variety of life processes (Zhu et al., 2021b; Zong et al., 2021). Dubs form a large family of proteases that can be divided into ubiquitin C-terminal hydrolases (UCHs), Machado-Joseph (MJD), ubiquitin-specific processing enzymes (UBPs), ovarian tumor-related proteases (OTU), and Jab1/MPN domain associated metalloisopeptidase (JAMM) (Ning et al., 2020). Dub diversity leads to their specificity in cutting Ub chains, *e.g*., Dubs only targeting K48-linked Ub chains or K63-linked Ub chains (Aalto et al., 2022). In the IMD pathway, Dredd and Imd proteins are key factors, and their ubiquitination levels are crucial for the downward transmission of the IMD pathway. Dredd has cysteine hydrolase activity, and Dredd hydrolase activity can only be manifested following ubiquitination (Russell et al., 2021). K63-linked ubiquitination also activates the Imd, which becomes a scaffold for the recruitment of downstream molecules, and is an essential link for the activation of the IMD pathway (Cammarata-Mouchtouris et al., 2022). However, the K48-linked ubiquitination of Imd and its mechanism remain largely unexplored.

In this study, we investigated an OTU Dubs family gene, *CG4968*, in *Drosophila*. Using molecular screens such as knockdown and overexpression, we found that CG4968 is dependent on the OTU domain in order to positively regulate IMD innate immunity. Additionally, CG4968 flies reduced the proliferation of Gram-negative pathogenic bacteria and were resistant to external pathogenic infections. We also found that CG4968 interacted with Imd proteins and mediated K48-linked deubiquitination of Imd through the OTU domain using *in vivo* ubiquitination analysis. In conclusion, our study reveals a positive regulatory mechanism of the IMD pathway involving CG4968 and Imd protein. It provides new evidence in the study of innate immunity of *Drosophila*.

## Materials and Methods

### *Drosophila* strain and husbandry

All of the *Drosophila* utilized in this study were raised on standard *Drosophila* medium, which consisted of agar and maize meal. *Drosophila* were reared at 25 °C and 12 h of alternating light and dark. The control and host for P-element-mediated transformation was the *w*^*1118*^ strain. In this investigation, the following Gal4 lines and transgenes were employed: (1) P{*UAS-CG4968*^*KD*^} obtained from Tsinghua *Drosophila* Centre (No. TH04864.N); (2) P{*ppl-Gal4*} obtained from Bloomington Stock Center; and (3) P{*UAS-CG4968*^*OE*^} and P{*UAS-CG4968*^*ΔOTU*^}, in which the full-length CG4968 and CG4968^ΔOTU^ were placed under the control of the UASp promoter. Among these, P{*ppl-Gal4*} could drive the specific high expression of genes in the fat bodies of adult flies.

### RNAi knockdown assays in S2 cells

We conducted synthesis of dsRNA using the T7 *in vitro* transcription kit (QIAGEN, Hilden, Germany) and RNase-Free throughout the experiment. All dsRNAs were synthesized according to the standard protocol. The specific primers of dsRNA were first designed using Primer Premier 5.0 software, and polymerase chain reaction amplification was then carried out. The amplified fragment was subjected to T7 transcription *in vitro* to synthesize dsRNA. At a density of 1 × 10^6^ cells per ml, S2 cells were harvested, diluted into fresh medium, and immediately treated with dsRNA. S2 cells were treated with dsRNA for 48 h, and then transfected with related plasmids. Primers used for dsRNA production are listed in Table S1.

### *Drosophila* S2 cell transfection and luciferase reporter assays

*Drosophila* S2 cells were kept in our laboratory for long-term storage (Hua et al., 2022). At 27 °C, S2 cells were grown in insect medium (Gibco, Waltham, MA, USA) with 10% fetal bovine serum (Hyclone, Logan, UT, USA). All of the cells utilized in this investigation were transfected using Lipofectamine 2000 (Invitrogen, Waltham, MA, USA). Transfection was divided into two Groups: in Group 1, plasmid and Opti-MEM were mixed at a ratio of 1 μg:50 μl, and in Group 2, plasmid and Lipofectamine 2000 were mixed at a ratio of 1 μg:2 μl. Then, the liquids of Group 1 and Group 2 were fully mixed. The S2 cells were collected 48 h after plasmid transfection.

For the luciferase reporter assays, we used the Drosomycin-luciferase or Attacin-luciferase constructs in which the luciferase coding sequence was put under the Drosomycin or Attacin promoter, Toll, and IMD signaling reporter assays in S2 cells. The procedure used to measure Firefly luciferase and Renilla luciferase was as follows. In order to perform luciferase experiments using a luciferase assay reagent (Promega), the S2 cells were lysed with 50 ml of Passive lysis buffer (Promega), according to the manufacturer’s instructions, and 20 μl cell lysate of each sample was placed in a 96-well plate containing luciferase substrate. Finally, the ratio of Firefly luciferase and Renilla luciferase was calculated, and three biological repelicates were performed.

### qRT-PCR assays

All samples were extracted using Trizol Reagent (Invitrogen, Waltham, MA, USA) to determine total RNA. In the following step, cDNA was synthesized using the first-strand cDNA synthesis kit (Transgen, Beijing, China) according to the manufacturer’s instructions. On a Light Cycler 480, qRT-PCR was performed in triplicate using SYBR Green Master Mix (Thermo Fisher Scientific, Waltham, MA, USA). The PCR reaction procedure was as follows: denaturation at 95 °C for 30 s, annealing at 55 °C for 30 s, and extension at 72 °C for 30 s for a total of 35 cycles. Template concentrations were normalized to an endogenous reference, Rp49. The data used in the detection were all analyzed using the 2^−ΔΔct^ method. Three biological replicates were performed. The primers for qRT-PCR were designed using Primer Premier 5.0 software. The primers for qRT-PCR production are shown in Table S2.

### Western blotting and co-immunoprecipitation (Co-IP)

We collected cell samples and lyse in buffer (150 mM NaCl, 50 mM Tris-HCl, pH 7.5, 10% glycerol, 0.5% Triton X-100, 10 μg/ml aprotinin, 10 μg/ml leupeptin, and 1 mM phenylmethylsulfonyl fluoride) for 40 min. The processed protein samples were put onto a protein gel for SDS-PAGE analysis, which was then run at 100 V for 30 min followed by 150 V for 30 min. The protein samples were then electrotransferred on ice using PVDF membranes (Thermo Fisher Scientific, Waltham, MA, USA) at a constant voltage of 100 V, blocked with skim milk, and then washed with PBST. Primary antibodies were incubated overnight at 4 °C and secondary antibodies were incubated at room temperature for 2 h. Both were washed with PBST. The following antibodies were used for Western blot: mouse anti-His (1:2,000; HRP), mouse anti-GST (1:3,000; Abcam, Cambridge, UK), mouse anti-Flag (1:3,000; Sigma-Aldrich, St. Louis, MO, USA), rabbit anti-Myc (1:2,000; Medical & Biological Laboratories, Nagoya, Japan), mouse anti-Tubulin (1:2,000; Cowin, Cambridge, MA, USA), rabbit anti-HA (1:1,000; Medical & Biological Laboratories, Nagoya, Japan). Goat anti-mouse IgG H&L (1:2,500; Abcam, Cambridge, UK) and goat anti-rabbit IgG H&L (1:2,500; Abcam, Cambridge, UK) were the secondary antibodies utilized for Western blot.

For the Co-IP experiment, cells were collected after plasmid transfection for 48 h and lysed in buffer-1 (150 mM NaCl, 50 mM Tris-HCl, pH 7.5, 10% glycerol, 0.5% Triton X-100, 10 μg/ml aprotinin, 10 μg/ml leupeptin, and 1 mM phenylmethylsulfonyl fluoride) for 40 min. Cell lysate was combined with Anti-Flag affinity gel (Sigma) at 4 °C for 4 h. Buffer-2 (50 mM Tris-HCl, pH 7.5, 150 mM NaCl, 5 mM EDTA, and 0.5% Igepal CA-6030) was used to wash the immune complexes three times for 20 min each time. Finally, the corresponding antibody was used for immunoblotting.

### Protein purification and GST pull-down assays

The *Escherichia coli* (*E.coli*) strain BL21 was used to produce and purify the GST-tagged or His-tagged proteins. Isopropyl-beta-D-thiogalactopyranoside (IPTG) was added to increase the protein expression final dosage of 1 mM into BL21 bacterial medium when the culture’s OD600 reached 0.5–0.7, then inducted at 16 °C in a 300 rpm shaker overnight. Bacteria cells were pelleted and resuspended in lysis buffer (25 mM Tris-HCl, pH 7.5, 100 mM NaCl, and 2 mM EDTA). Each sample was sonicated for 40 min using an EpiShearTM Probe Sonicator (pulse 6s on, 10s off, 40% amplitude) to disturb the integrity of the cells. After centrifuging the sample at 10,000 rpm for 40 min at 4 °C, the protein concentration was extracted from the supernatant. For the purification of GST-tagged or His-tagged proteins, BeyoGoldTM His-tag Purification Resin (Beyotime, Nantong, China) or Glutathione Sepharose (Sigma, St. Louis, MO, USA) were employed, respectively. Finally, loading buffer was added to each sample (1 mg), and the Bradford assay was performed. The appropriate proteins were treated with Glutathione Sepharose at 4 °C for 4 h to perform the GST pull-down. The samples were then Western blot tested after being washed three times with PBS and 1% TritonX-100 for a total of 1 h.

### *In vivo* ubiquitination assay

Transfected S2 cells were collected and lysed in buffer A (50 mM Tris-HCl, pH 7.5, 150 mM NaCl, 0.5% Nonidet P-40, 10% glycerol and 1% SDS) for 40 min. After heating at 95 °C for 10 min and mixed thoroughly with buffer B (50 mM Tris-HCl, pH 7.5, 150 mM NaCl, 0.5% Nonidet P-40, and 10% glycerol). After ultrasonic treatment of the lysate (pulse 4s on, 6s off, 30% amplitude), Anti-Flag beads (Sigma, St. Louis, MO, USA) were used to bind the lysate at 4 °C for 6 h. The conjugate was washed three times for 20 min each with washing buffer (50 mM Tris-HCl, pH 7.5, 500 mM NaCl, 0.5% Nonidet P-40, and 10% glycerol). Western blot analysis was then performed to detect the ubiquitination pattern of the specified protein and analysed using Image J software.

### Survival and bacterial CFU assays

Bacterial infections were carried out using the following method. A small needle dipped in a concentrated overnight culture of bacteria was used to puncture 6-day-old adult flies. Flies were transferred to a new medium each day after infection. In the survival study score, 30 flies per group were kept in the same environment. The number of flies that survived was counted daily. Flies that died within 3 h of bacterial infection were not included in this study. For the CFU counting test, 10 infected flies were taken at the appropriate time after infection and crushed in PBS. We plated 100 μl of each dilution on LB agar plates. After overnight growth in an incubator at 30 °C, bacterial colonies were counted.

### Statistical analysis

GraphPad Prism 8 was used to perform all statistical analyses. The mean and standard errors of the data were displayed. Except for lifetime assays, which employed the log-rank test for statistical analysis, significance was assessed using two-tailed Student’s t tests. *p* < 0.05 was considered statistically significant (ns, not significant; *p < 0.05; **p < 0.01; ***p < 0.001).

## Results

### CG4968 is a positive regulator of the IMD pathway

**Figure 1:**
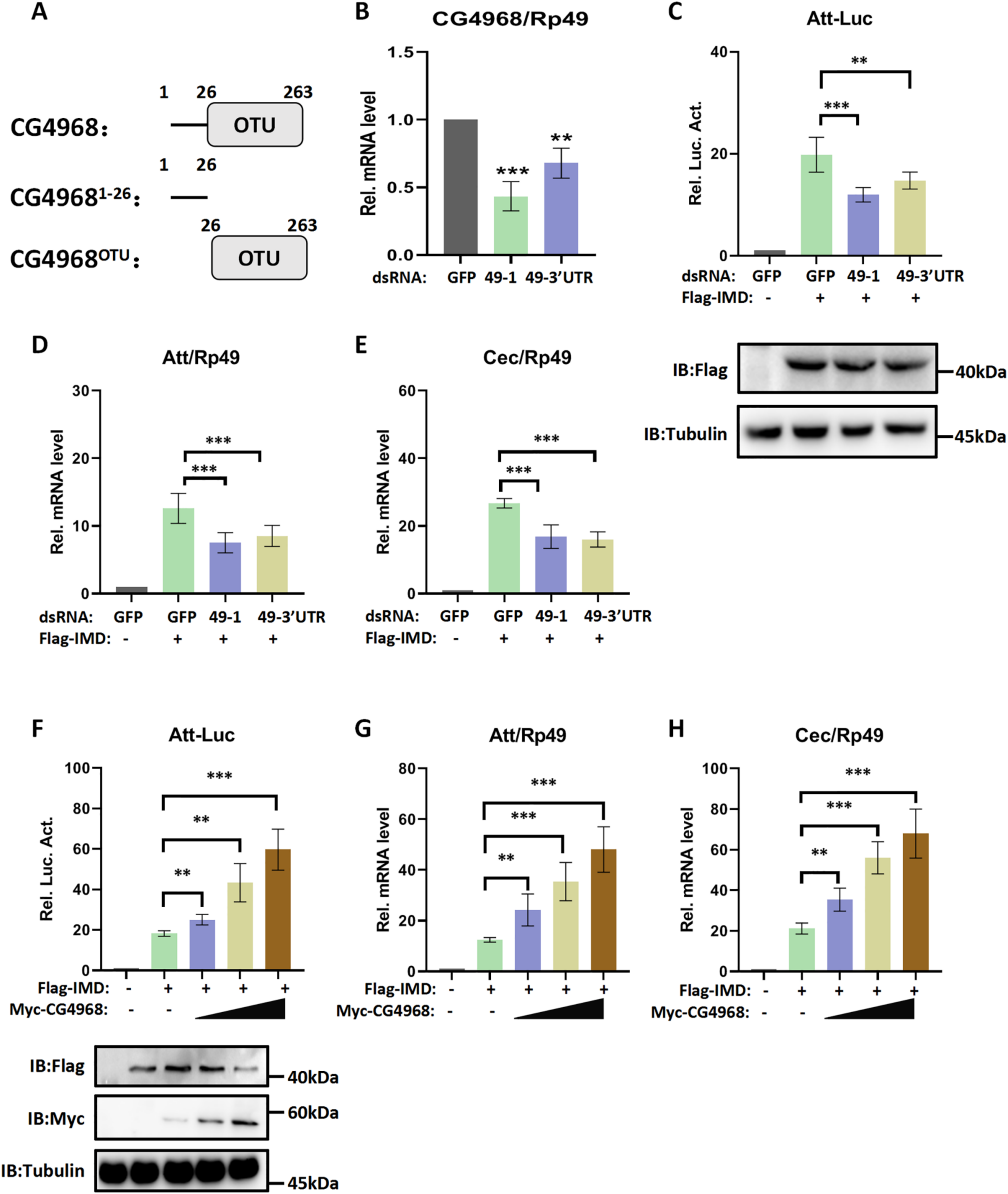
Deubiquitinating enzyme (Dub) CG4968 positively regulates IMD immune pathway activity in *Drosophila* S2 cells. (A) Domain architecture of CG4968. (B) qRT-PCR assays for dsRNA 49-1 and dsRNA 49-3′UTR knockdown of CG4968 expression in *Drosophila* S2 cells, Rp49 was used as an internal control. (C) *Drosophila* S2 cells were treated with the dsRNA GFP, dsRNA 49-1, and dsRNA 49-3′UTR for 48 h. The reporter plasmid and associated expression vector were transfected into dsRNA knockdown cells. The cells were lysed for Luciferase detection and Western blot 36 h later. (D–E) *Drosophila* S2 cells were treated with the corresponding dsRNA for 48 h. Transfection of relevant expression vectors into dsRNA knockdown cells. qRT-PCR assay to check transcriptional levels of Attacin (Att) (D) and Cecropin (Cec) (E) 36 h later, and Rp49 was used as an internal control. (F) The Myc-CG4968 at 1, 2, and 4 μg concentrations was transfected with Flag-IMD plasmid and reporter plasmid into *Drosophila* S2 cells in a gradient. The cells were lysed for Luciferase detection and Western blot 36 h later. (G–H) The Myc-CG4968 at 1, 2 and 4 μg concentrations was transfected with Flag-IMD plasmid. qRT-PCR assay to check transcriptional levels of Att (G) and Cec (H) 36 h later, and Rp49 was used as an internal control. (B–H) Error bars represent SD (*n* = 3). The two-tailed Student’s t test was used to analyze statistical significance. **p < 0.01, ***p < 0.001.

Recent research has found that Dubs play an important role in regulating the immune pathway in *Drosophila* (Hua et al., 2022; Ji et al., 2019). Our excavation of the *Drosophila* genome resulted in the discovery of a functionally undefined Dub gene, *CG4968* (Gene ID: 34384). Analysis of the domain of CG4968 through the Flybase online website (https://flybase.org/) showed that it has an OTU domain, indicating that CG4968 is a typical member of the OTU Dubs family (Fig. 1A). To determine whether CG4968 is involved in the regulation of innate immunity in *Drosophila*, we first designed dsRNAs targeting the CG4968 coding sequence and the 3′-untranslated region (dsRNA 49-1, dsRNA 49-3′UTR). The qRT-PCR results showed that both of the two groups of dsRNAs significantly knocked down the expression of *CG4968* in *Drosophila* S2 cells (Fig. 1B). We then constructed an Attacin-Luciferase reporter system (Att-Luc), and the firefly luciferase gene was placed under the control of the Attacin promoter. The IMD pathway was activated by expressing the active form of IMD, resulting in the activation of luciferase expression driven by the Attacin gene promoter, and showed dosage and temporal trends (Fig. S1). Att-Luc reporter assay revealed that the knockdown of *CG4968* expression in S2 cells significantly reduced Att-Luc activity activated by IMD overexpression (Fig. 1C). Additionally, we examined the effect of the knockdown of *CG4968* in S2 cells on the expression of antimicrobial peptides (AMPs) of the IMD immune pathway. qRT-PCR results showed that the knockdown of *CG4968* significantly reduced the expression of AMPs *Attacin* (*Att*) and *Cecropin* (*Cec*) downstream of the IMD pathway compared to the control group, which was similar to the results of the Att-Luc reporter assay (Figs. 1D and 1E).

To further explore the association of CG4968 with the IMD pathway, *CG4968* was overexpressed in S2 cells. The results showed that *CG4968* overexpression significantly increased Att-Luc activity induced by the active form of IMD (Fig. 1F). Additionally, the expression levels of *Att* and *Cec* in the IMD pathway increased significantly with the increase of *CG4968* concentration (Figs. 1G and 1H). The above results suggested that the Dub CG4968 positively regulated the IMD pathway in S2 cells.

### CG4968 regulates IMD immune pathway *via* the OTU domain

**Figure 2:**
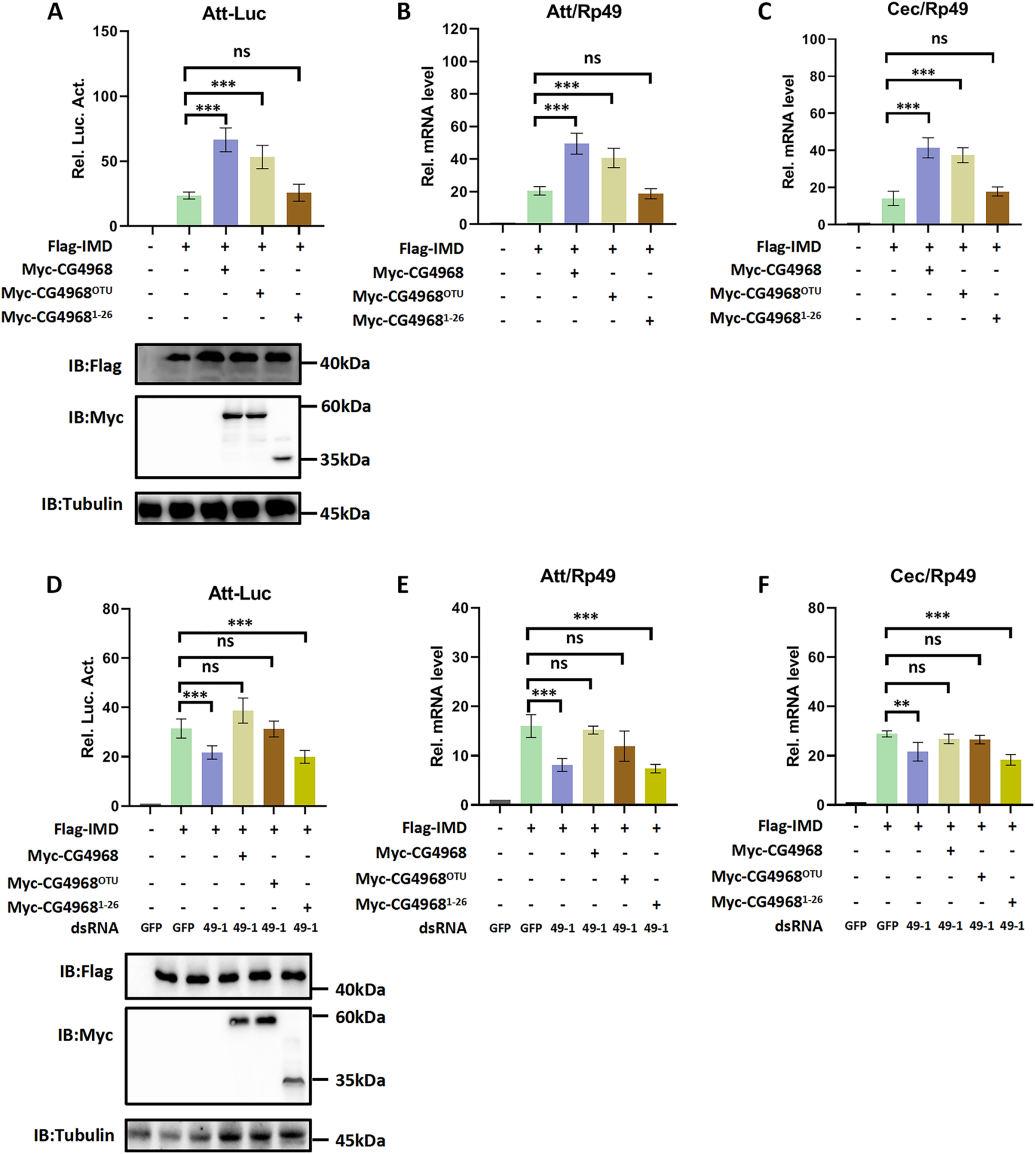
The OTU domain is key to the positive regulation of the IMD immune pathway by CG4968. (A) The above related plasmids were transfected with the reporter plasmid into *Drosophila* S2 cells; 36 h later, the cells were lysed for Luciferase detection and Western blot. (B–C) The above related plasmids were transfected; 36 h later, qRT-PCR assay to check transcriptional levels of Att (B) and Cec (C), and Rp49 was used as an internal control. (D) *Drosophila* S2 cells were treated with the corresponding dsRNA GFP and dsRNA 49-1 for 48 h. The reporter plasmid and associated expression vector were transfected into dsRNA knockdown cells. The cells were lysed for Luciferase detection and Western blot 36 h later. (E–F) After transfection of the dsRNA GFP and dsRNA 49-1 into *Drosophila* S2 cells for 48 h, the relevant plasmids were transfected into dsRNA-treated S2 cells for 36 h. qRT-PCR assay to check transcriptional levels of Att (E) and Cec (F), and Rp49 was used as an internal control. (A–F) Error bars represent SD (*n* = 3). The two-tailed Student’s t test was used to analyze statistical significance. ns, not significant, **p < 0.01, ***p < 0.001.

In order to gain more insight into the mechanism of CG4968 regulating the IMD pathway, two truncated forms of CG4968 expression plasmids (CG4968^1-26^ and CG4968^OTU^) were constructed based on domain analysis (Fig. 1A). The CG4968, CG4968^1-26^, and CG4968^OTU^ each transfected *Drosophila* S2 cells. The Att-Luc reporter assay showed that the expression of *CG4968* and *CG4968*^*OTU*^ significantly enhanced induced Att-Luc activity compared to the control group. After transfection with CG4968^1-26^, Att-Luc activity was not significantly different from that of the control group (Fig. 2A). Also, the qRT-PCR results showed that the expression of *CG4968* and *CG4968*^*OTU*^ in S2 cells significantly elevated the expression of *Att* and *Cec* of the IMD pathway compared to that of the control. However, the expression of *CG4968*^*1-26*^ showed no difference in the expression of *Att* and *Cec* compared to that of the control group (Figs. 2B and 2C).

In addition, we performed rescue experiments to explore the role of the CG4968 OTU domain in the positive regulation of the IMD pathway. S2 cells were first treated with dsRNA 49-1 to attenuate IMD pathway activity and then transfected with plasmids CG4968, CG4968^1-26^, and CG4968^OTU^ to determine the rescue effects of CG4968, CG4968^1-26^, and CG4968^OTU^ on the decrease of IMD pathway signal activity. The results showed that during the expression of dsRNA 49-1, the activity of Att-Luc decreased due to the downregulation of *CG4968* expression. At the same time during the expression of *CG4968* and *CG4968*^*OTU*^, Att-Luc activity was significantly increased compared with the control group. However, *CG4968*^*1-26*^ did not significantly elevate Att-Luc activity reduced by knockdown of *CG4968* expression due to its lack of OTU domain (Fig. 2D). We also detected the expression of *AMPs* in each group using qRT-PCR and found that the expression of *CG4968*^*OTU*^ effectively rescued the decreased expression of the IMD pathway *Att* and *Cec* caused by the knockdown effect of dsRNA 49-1, similarly to that of the expression of *CG4968*. However, the expression of *CG4968*^*1-26*^ did not have a rescue effect on the decline of the IMD pathway *AMPs* compared to the expression of *CG4968*^*OTU*^ (Figs. 2E and 2F). This suggests that the OTU domain of CG4968 is indispensable for its positive regulation of the *Drosophila* IMD pathway.

### CG4968 is non-essential for the Toll immune pathway

**Figure 3:**
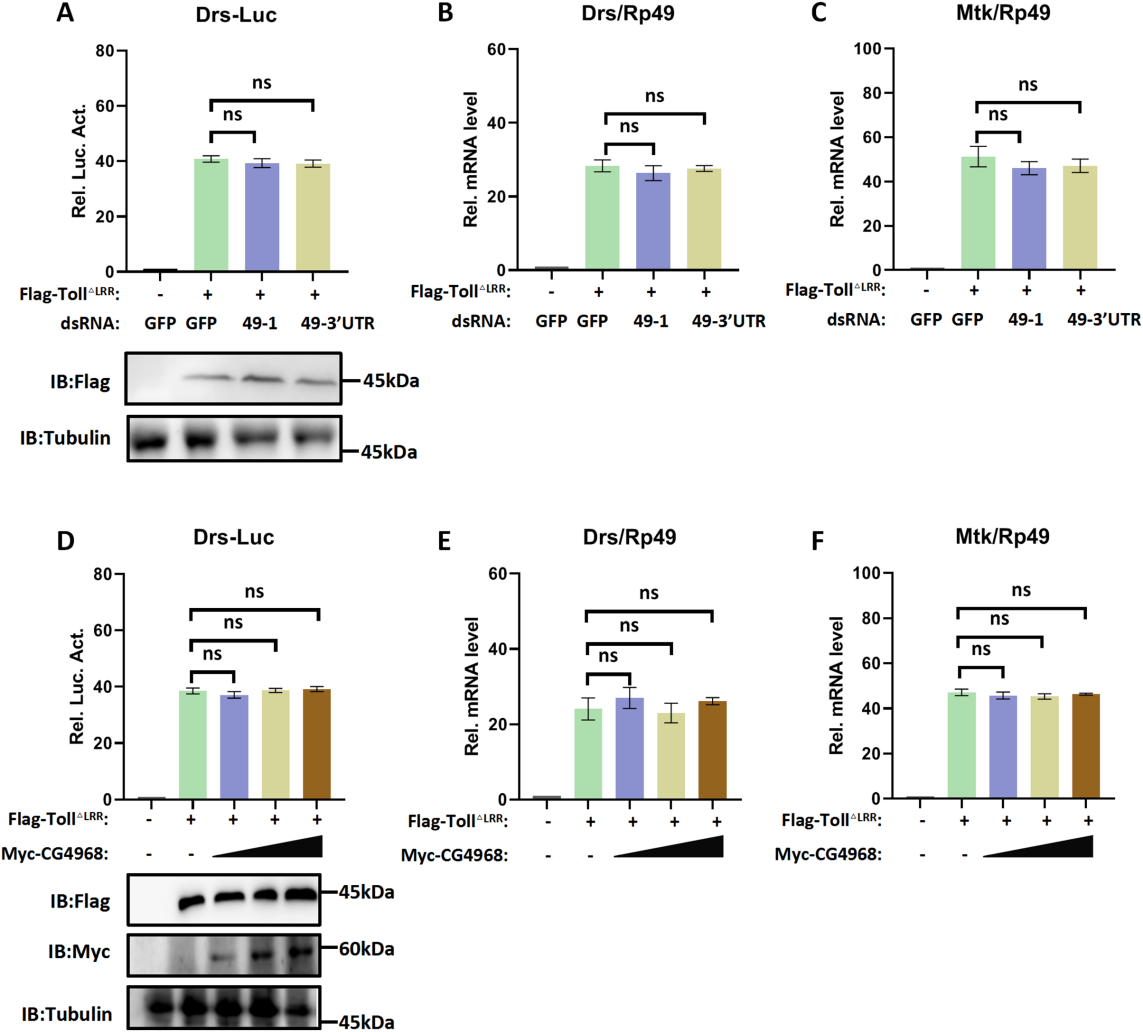
CG4968 does not regulate the *Drosophila* S2 cells Toll immune pathway. **(**A) *Drosophila* S2 cells were treated with the dsRNA GFP, dsRNA 49-1, and dsRNA 49-3′UTR for 48 h. The reporter plasmid and associated expression vector were transfected into dsRNA knockdown cells. The cells were lysed for Luciferase detection and Western blot 36 h later. (B–C) *Drosophila* S2 cells were treated with the corresponding dsRNA for 48 h. Transfection of relevant expression vectors into dsRNA knockdown cells. qRT-PCR assay to check transcriptional levels of Drosomycin (Drs) (B) and Metchnikowin (Mtk) (C) 36 h later, and Rp49 was used as an internal control. (D) The Myc-CG4968 at 1, 2, and 4 μg concentrations was transfected with Flag-Toll^ΔLRR^ plasmid and reporter plasmid into *Drosophila* S2 cells in a gradient. The cells were lysed for Luciferase detection and Western blot 36 h later. (E–F) The Myc-CG4968 at 1, 2, and 4 μg concentrations was transfected with Flag-IMD plasmid. qRT-PCR assay to check transcriptional levels of Mtk (E) and Drs (F) 36 h later, and Rp49 was used as an internal control. (A–F) Error bars represent SD (*n* = 3). The two-tailed Student’s t test was used to analyze statistical significance. ns, not significant.

Given the regulatory role of CG4968 on the IMD pathway, we also used the Drosomycin promoter-driven luciferase reporter system (Drs-Luc) to explore the regulatory role of CG4968 on the Toll immune pathway. The expression of Drs-Luc activated overexpression of the active form of Toll^ΔLRR^ and showed dosage and temporal trends (Fig. S2). We found that knockdown of *CG4968* expression in S2 cells had no significant effect on the increased expression of Toll^ΔLRR^ activated Drs-Luc activity (Fig. 3A). We also analysed whether knockdown of *CG4968* in S2 cells affected the expression levels of the *AMPs Drosomycin* (*Drs*) and *Metchnikowin* (*Mtk*) of the Toll immune pathway. The results showed that the knockdown of *CG4968* did not affect the expression of *Drs* and *Mtk* compared with the control group (Figs. 3B and 3C).

Meanwhile, we used overexpressed *CG4968* in S2 cells at a concentration gradient to investigate whether overexpression of CG4968 affected the Drs-Luc activity activated by Toll^ΔLRR^ and the expression level of AMPs of the Toll immune pathway. The Drs-Luc reporter assay showed that the gradient overexpression of *CG4968* in S2 cells did not affect Drs-Luc activity compared to the control (Fig. 3D). Similarly, overexpression of *CG4968* had no effect on the expression of the Toll immune pathway *Drs* and *Mtk* (Figs. 3E and 3F). Based on the above results, our study shows that although CG4868 can positively regulate the IMD pathway, it has no effect on the transduction of the Toll immune pathway.

### CG4968 regulates the K48-linked deubiquitination of Imd *via* the OTU domain

**Figure 4:**
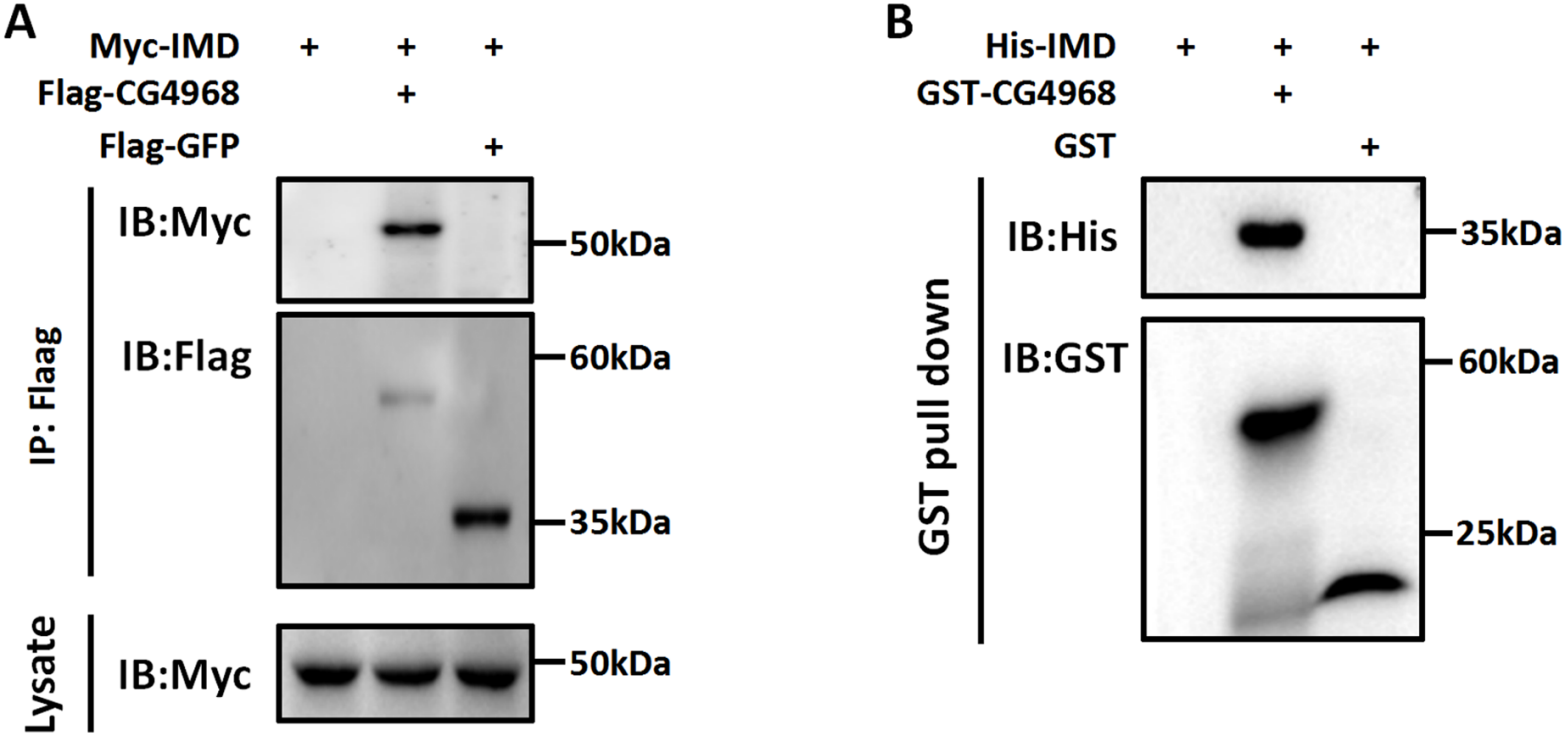
Interaction between CG4968 and Imd proteins. (A) The above relevant expression plasmids were transfected into S2 cells individually and after 48 h, cells were lysed using anti-Flag agarose beads for immunoprecipitation analysis, followed by Western blot analysis. (B) The purified His-tagged Imd protein was incubated with GST-tagged for 4 h, and then the samples were subjected to GST pull-down and Western blot analysis, with GST as the control.

Imd protein is an important connector protein that plays an upstream and downstream role in the IMD pathway (Russell et al., 2021). Our study found that IMD pathway activity was significantly higher in S2 cells cotransfected with IMD and CG4968 plasmids than with IMD plasmids alone (Figs. 1F–1H). To determine whether there is an interaction between CG4968 and Imd, Co-IP experiments were performed in S2 cells with co-transfected IMD and CG4968 plasmids (empty plasmids expressing GFP were used as controls). The Co-IP results showed a clear interaction between CG4968 and Imd (Fig. 4A). In order to further explore the interaction between CG4968 and Imd, we purified GST-tagged CG4968 and His-tagged Imd from *E. coli* (Fig. S3). As shown in the GST pull-down assays, we found a significant interaction between purified GST-CG4968 and His-Imd (Fig. 4B). This further confirms the interaction between CG4968 and Imd.

**Figure 5:**
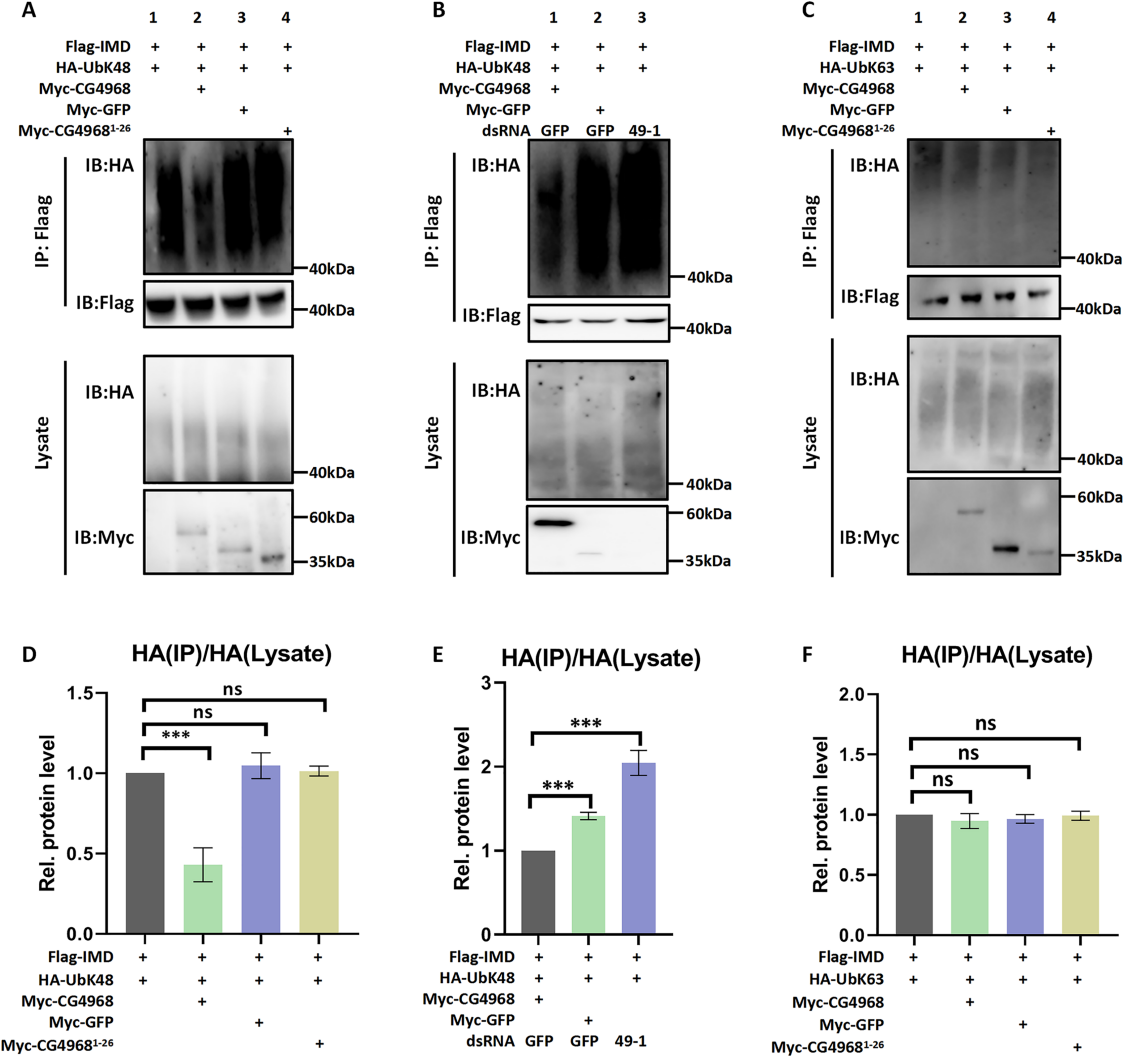
CG4968 inhibits K48-linked ubiquitination of Imd proteins through the OTU domain. The above related groups of expression plasmids (HA-Ub is HA-UbK48) were transfected into *Drosophila* S2 cells for 48 h and then subjected to ubiquitination analysis. (D) Densitometric analysis of quantitative Imd ubiquitination levels (A) is shown. (B, E) *Drosophila* S2 cells were treated with dsRNA GFP and dsRNA 49-1 for 36 h and then transfected with the expressed plasmids for 48 h and then subjected to ubiquitination analysis. (E) Densitometric analysis of quantitative Imd ubiquitination levels (B) is shown. (C, F) The above related groups of expressed plasmids (HA-Ub is HA-UbK63) were transfected into *Drosophila* S2 cells for 48 h and then subjected to ubiquitination analysis. (F) Densitometric analysis of quantitative Imd ubiquitination levels (C) is shown. (D–F) Error bars represent SD (*n* = 3). The two-tailed Student’s t test was used to analyze statistical significance. ns, not significant, ***p < 0.001.

To explore which type of deubiquitination of Imd is mediated by CG4968, we used a series of ubiquitin mutants for analysis. Our ubiquitination experiments showed that CG4968 overexpression in S2 cells significantly reduced ubiquitination modifications associated with the K48-linkage of Imd (all lysines in ubiquitin except K48 were mutated to arginine) compared to the control group’s expression of GFP. However, the expression of CG4968^1-26^ had no effect on the K48-linked ubiquitination modifications of Imd compared to the control (Figs. 5A and 5D). To further confirm that CG4968 regulates the K48-linked deubiquitination modification of Imd, we treated S2 cells with dsRNA 49-1 to explore the effects of CG4968 knockdown and overexpression in S2 cells on the K48-linked deubiquitination modification of the Imd. The results showed that overexpression of CG4968 significantly reduced the K48-linked ubiquitination modification of the Imd compared to the control but knockdown of CG4968 expression in S2 cells significantly elevated the level of K48-linked ubiquitination modification of Imd (Figs. 5B and 5E). We also tested whether CG4968 affected the level of K63-linked (all lysines in ubiquitin were mutated to arginine except for K63) ubiquitination modification of Imd. *In vivo* ubiquitination experiments showed that CG4968 had no effect on the level of K63-linked ubiquitination modification of the Imd compared to controls (Figs. 5C and 5F).

This suggests that CG4968 significantly inhibited the K48-linked ubiquitination modification of the Imd, with the OTU domain acting as the key to CG4968 inhibiting the K48-linked ubiquitination modification of Imd.

### *Drosophila* CG4968 executes its physiological function *via* OTU domain

**Figure 6:**
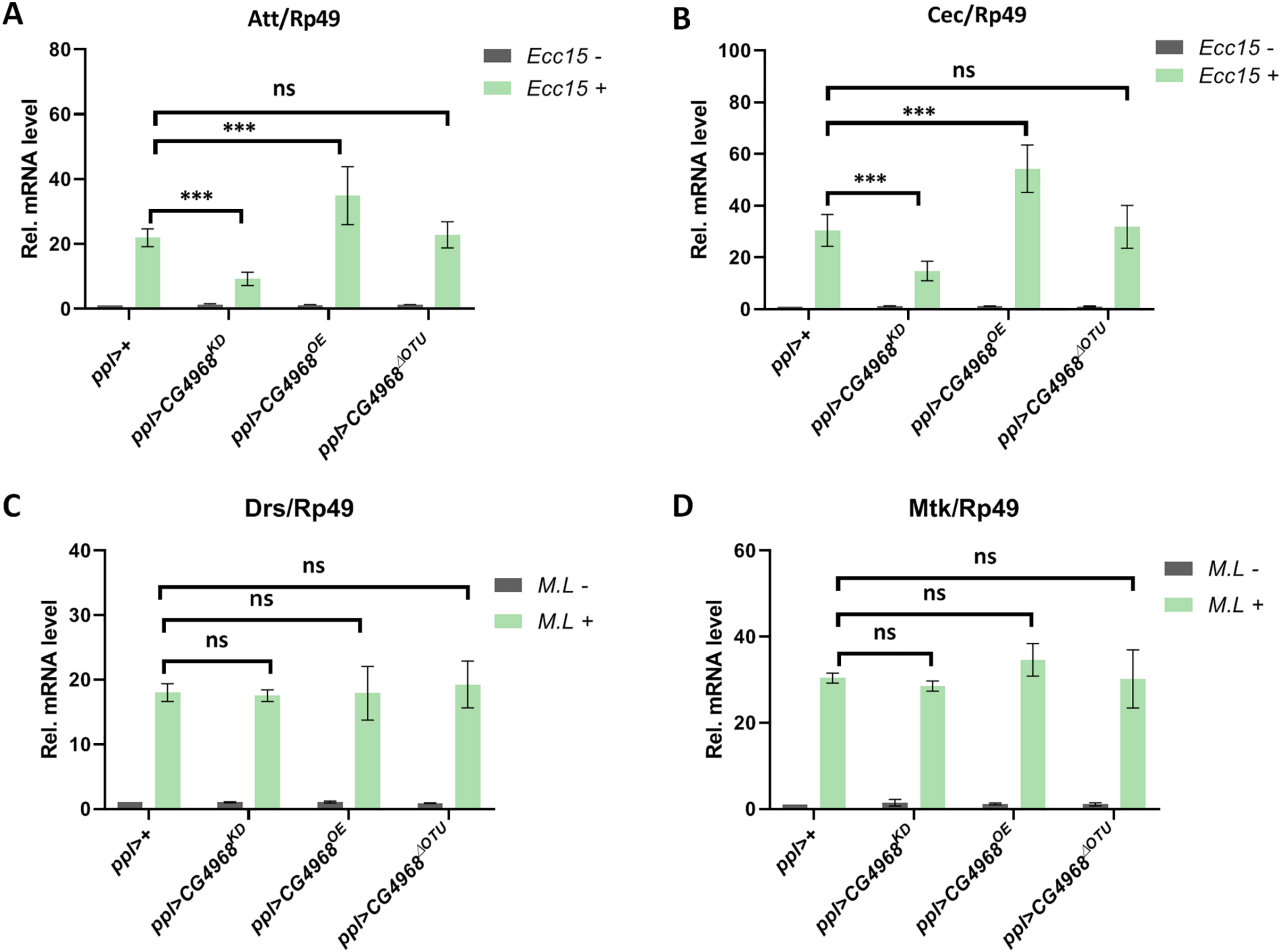
CG4968-dependent OTU domain positively regulates the IMD immune pathway in flies. (A, B) Male adults including *ppl>+*, *ppl>CG4968^KD^*, *ppl>CG4968^OE^*, and *ppl>CG4968^ΔOTU^* were either infected with *Erwinia carototovora ASP 15* (*Ecc15 ^+^*) or not infected (*Ecc15 ^−^*). Twelve h later, flies were harvested and lysed. qRT-PCR assay to check transcriptional levels of Att (A) and Cec (B), and Rp49 was used as an internal control. (C, D) Male adults including *ppl>+*, *ppl>CG4968^KD^*, *ppl>CG4968^OE^*, and *ppl>CG4968^ΔOTU^* were either infected with *Micrococcus luteus* (*M.L ^+^*) or not infected *(M.L ^−^*). Twelve h later, flies were harvested and lysed. qRT-PCR assay to check transcriptional levels of Mtk (C) and Drs (D), and Rp49 was used as an internal control. (A–D) Error bars represent SD (*n* = 3). The two-tailed Student’s t test was used to analyze statistical significance. ns, not significant, *** p < 0.001.

Next, to investigate the physiological function of CG4968 *in vivo*, we constructed the overexpression of CG4968 flies P{*Uasp-CG4968*^*OE*^} and CG4968^1-26^ flies P{*Uasp-CG4968*^*ΔOTU*^} and obtained the knockdown of CG4968 expression flies P{*Uasp-CG4968*^*KD*^}. Because flies’s fat body serves as an important organ (Ramirez-Corona et al., 2021), we crossed the flies with P{*ppl-gal4*}, which is a fat body-specific driver. Characterization of transgenic flies revealed that P{*ppl-gal4*} effectively reduced and increased the expression of CG4968 in the fat body (Fig. S4). We infected 6-day-old control and transgenic flies with *Erwinia carototovora ASP 15* (*Ecc15*), a commonly-used bacterium that can induce the response of the IMD pathway and the expression of AMPs (Hua et al., 2022). The qRT-PCR results showed that the expression of *Att* and *Cec* of the IMD pathway in *ppl>CG4968*^*KD*^ was significantly lower than that in the control *ppl>+* after infection with *Ecc15*. The expression of *Att* and *Cec* was significantly higher in *ppl>CG4968*^*OE*^ after infection with *Ecc15* compared to the control *ppl>+*. Interestingly, we also detected no significant difference in the expression of *Att* and *Cec* in *ppl>CG4968*^*ΔOTU*^ compared to *ppl>+* due to the deletion of the OTU domain (Figs. 6A and 6B). This suggests that CG4968 is dependent on the OTU domain for positive regulation of the IMD pathway in flies.

Similarly, we used the Gram-positive bacterium *Micrococcus luteus* (*M.L*) to infect 6-day-old flies to activate Toll immune pathway responses (Ji et al., 2014). qRT-PCR results showed that the expression of the Toll immune pathways *Mtk* and *Drs* was not significantly different in transgenic flies (*ppl>CG4968*^*KD*^, *ppl>CG4968*^*OE*^, *ppl>CG4968*^*ΔOTU*^) compared to *ppl>+* after infection with *M.L* (Figs. 6C and 6D). This suggests that CG4968 has no effect on the Toll immune pathway in flies.

**Figure 7:**
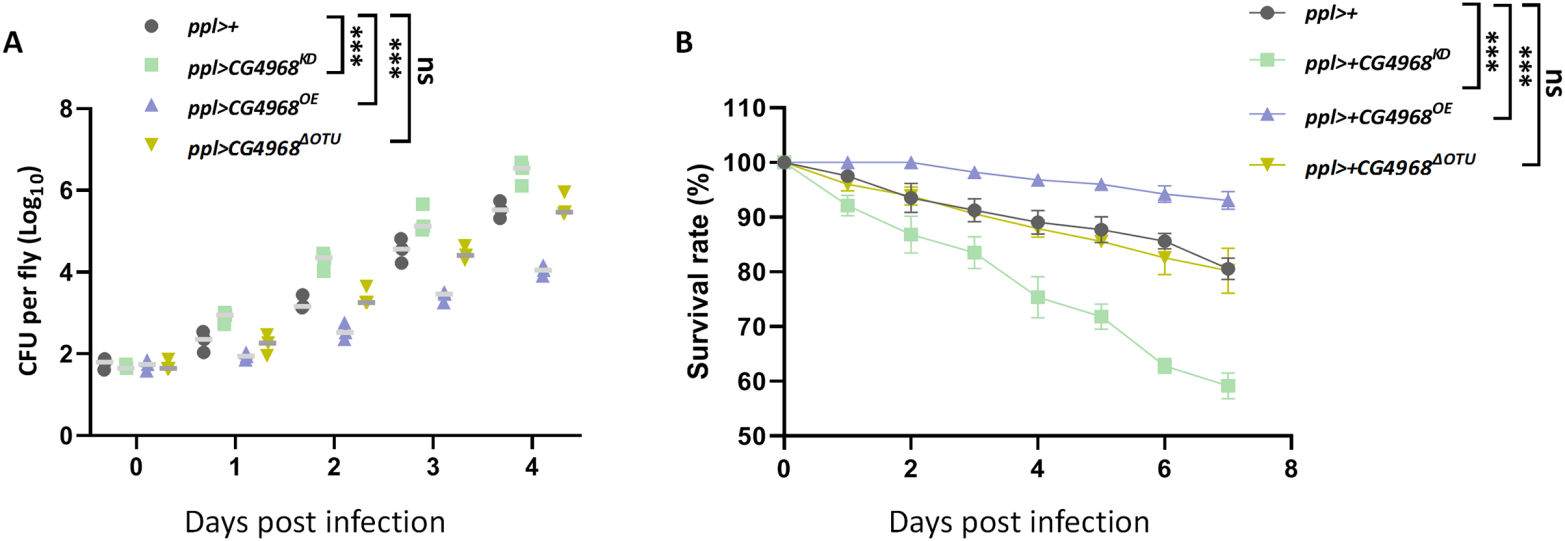
CG4968 relies on the out domain to enhance the ability of flies to defend itself against external pathogens. (A) Male adults including *ppl>+*, *ppl>CG4968^KD^*, *ppl>CG4968^OE^*, and *ppl>CG4968^ΔOTU^* were pricked with a needle previously dipped in *Serratiamarcescens* (*S.M*). Detection of bacterial proliferation at different times. (B) Male adults including *ppl>+*, *ppl>CG4968^KD^*, *ppl>CG4968^OE^*, and *ppl>CG4968^ΔOTU^* were pricked with S.M. Daily counts of fly survival. (A–B) Error bars represent SD (*n* = 3). The LogRank test in the PASW Statistics 18 software was used to analyze statistical significance. ns, not significant, ***p < 0.001.

To gain more insight into the physiological function of CG4968 *in vivo*, we examined the role of CG4968 in resistance to an exogenous pathogenic infestation in flies. Gram-negative pathogenic bacteria *Serratia marcescens* (*S. marcescens*) were used to infect *ppl>CG4968*^*KD*^, *ppl>CG4968*^*OE*^, and *ppl>CG4968*^*ΔOTU*^. The effect of CG4968 on the growth of *S. marcescens* bacteria in host flies was examined using bacterial load assay. When infected with *S. marcescens* pathogenic bacteria, the number of *S. marcescens* colonies in *ppl>CG4968*^*KD*^ was significantly higher than that of the control *ppl>+*, and the number of *S. marcescens* colonies in *ppl>CG4968*^*OE*^ was significantly lower than the control *ppl>+*. Due to the absence of the OTU domain, *ppl>CG4968*^*ΔOTU*^ showed no difference in the number of *S. marcescens* colonies in comparison to the control (Fig. 7A). Additionally, we measured the survival rate after infection of flies with *S. marcescens*. The results showed that the survival rate after infection of *ppl>CG4968*^*OE*^ flies with *S. marcescens* was significantly higher than the control *ppl>+*. On the contrary, the survival rate of *ppl>CG4968*^*KD*^ after infection with *S. marcescens* was significantly lower than that of the control group. The *ppl>CG4968*^*ΔOTU*^ survival rate did not differ compared to the control group (Fig. 7B). Taken together, we concluded that CG4968 inhibits the growth of Gram-negative pathogens by promoting the expression of AMPs of the IMD pathway, thereby increasing the survival rate of flies. It is noteworthy that the OTU domain is the key to the physiological function of CG4968 in flies.

## Discussion

In *Drosophila*, the IMD pathway is one of the major signaling cascades governing the secretion of a variety of AMPs upon pathogenic infection (Russell et al., 2021; Marra et al., 2021). To achieve this, the pivotal adaptor protein Imd must be modified by K63-linked ubiquitination (Ma et al., 2022). However, few studies have looked at whether the K48-linked ubiquitination of Imd is involved in the regulation of the IMD pathway. In this study, we report a positive regulator of the IMD pathway: the Dub CG4968. Functionally, CG4968 relies on the OTU domain to actively regulate the activity of IMD pathways. CG4968 flies showed good antibacterial activity against Gram-negative bacteria with an increased the survival rate. Mechanistically, Dub CG4968 mediated the K48-linked deubiquitination of Imd, but had no effect on the K63-linked deubiquitination.

The IMD pathway is named according to the immunodeficient phenotype of Imd gene mutation and is mainly responsible for resisting the infection of Gram-negative bacteria in nature (Kleino & Silverman, 2019). Following infection with Gram-negative bacteria, peptidoglycan recognition protein LC (PGRP-LC) and peptidoglycan recognition protein LR (PGRP-LR) in *Drosophila* recognize pathogenic bacteria and bind to the junction molecule Imd protein (Orlans et al., 2021). Imd, dFadd, and Dredd form the Imd/dFadd/Dredd complex. At the same time Uev1a, Bendless, and Effete form the E2 ubiquitin-conjugating enzyme complex, which acts in concert with dIap2 (E3 ubiquitin ligase) to activate Dredd ubiquitination. Dredd activation cleaves the Imd and the cleaved Imd is ubiquitinated by dIap2 to modify the K63 chain, thus triggering a signalling cascade (Kamareddine et al., 2018). Recent research has found that factors including Protein Phosphatase 4 (PP4), IIV-6, and dTrabid can negatively regulate the IMD pathway (Salem Wehbe et al., 2021; West et al., 2019; Fernando, Kounatidis & Ligoxygakis, 2014). In a molecular screening based on RNAi and overexpression *CG4968* in S2 cells, we found that the knockdown expression of *CG4968* significantly inhibited the activity of the IMD pathway, and the activity of the IMD pathway showed a dose-dependent trend with *CG4968*. The rescue result showed that the expression of the OTU domain saved the IMD pathway activity decreased after knockdown of *CG4968*. This preliminarily proves that CG4968 can actively regulate the IMD pathway. Further, the function of CG4968 *in vivo* was explored by constructing knockdown, overexpression, and OTU domain-deficient CG4968 flies. qRT-PCR results showed that after Gram-negative bacterial infestation, the expression of *AMPs* in *ppl>CG4968*^*KD*^ flies significantly decreased, while the expression of *AMPs* in *ppl>CG4968*^*OE*^ significantly increased. In particular, the expression of *AMPs* in *ppl>CG4968*^*ΔOTU*^ was not significantly different from *ppl>+*. Experiments in S2 cells and *Drosophila* strongly confirmed that CG4968 is a positive regulator of the IMD pathway, and the OTU domain played a key role in this process. Immediately following that, we explored the biological function of CG4968 *in vivo*. CFU and survival assays showed that CG4968 relied on its OTU domain to inhibit pathogen proliferation and improve survival by enhancing the ability of flies to resist exogenous pathogens. This further illustrates the biological role of CG4968 as a positive regulator of the IMD pathway. We found that when flies encountered exogenous pathogen infection, CG4968 significantly increased the expression of AMPs of the IMD pathway. CG4968 provided flies with the ability to resist pathogens.

There are seven lysine residue sites on ubiquitin proteins that can be modified *via* polyubiquitination, which exerts different physiological effects (Rabl, 2020). Among them, K48-linked ubiquitination mostly mediated the recognition and degradation of target proteins by the 26S proteasome, while K63-linked ubiquitination modifications mostly mediated signaling (Tracz & Bialek, 2021). Previous studies have shown that Dubs regulate innate immune responses by inhibiting K63-linked ubiquitination in *Drosophila*. A typical OTU Dub dTrabid rely on the OTU domain to inhibit the K63-linked ubiquitination of dTak1 and thus negatively regulate the IMD pathway (Hua et al., 2022). Dub Ubiquitin-specific protease 36 (dUSP36) can interact with Imd and negatively regulate the Imd pathway by inhibiting K63-linked ubiquitination of Imd (Thevenon et al., 2020). However, the ubiquitination of K48-linked has rarely been reported in the study of innate immunity of *Drosophila*. In this study, the type of CG4968-mediated deubiquitination of Imd was characterized using multiple ubiquitin mutants. Assays showed that CG4968 inhibited the K48-linked ubiquitination modification of Imd through the OTU domain, but did not affect the K63-linked ubiquitination of Imd. Our research shows that the de-ubiquitination modification of K48-linked ubiquitination plays the same important role as K63-linked ubiquitination in the innate immune regulation of *Drosophila*. However, in mammals, OTU Dub has been found to regulate the immune response by inhibiting the K48-linked ubiquitination of the target protein and increasing its stability. Dub OTUD5 can interact with the stimulator of interference genes (STING), promote the stability of STING through K48-linked ubiquitination, and play an important role in anti-virus and anti-tumor immunity (Guo et al., 2021). Dub OTUB1 can inhibit the K48-linked ubiquitination level of Programmed Death Ligand1 (PD-L1) and affect the immunosuppression of cancer by regulating the stability of PD-L1 (Zhu et al., 2021a). Dub OTUD3 played a negative regulatory role in tumor development by inhibiting the K48-linked ubiquitination of PTEN (Zhang et al., 2020). Based on previous research, our study shows that Dub CG4968 inhibits the K48-linked ubiquitination of Imd in *Drosophila* and positively regulates the IMD immune pathway by increasing the stability of Imd.

Previous studies on the ubiquitination modification of Imd revealed that the 137^th^ and the 153^th^ lysine residues were the main reaction sites for the K63-linked ubiquitination of Imd, which is essential for the downstream signaling transduction of the IMD pathway (Chen et al., 2017). However, the lysine site required for the K48-linked ubiquitination modification for Imd is still unknown, and should be determined using mutant and mass spectrometry.

In conclusion, we identified a Dub CG4968 in *Drosophila* that enhances the stability of Imd by targeting K48-linked deubiquitination through the OTU domain, and thus exerts a role in positively regulating the IMD pathway. This study provides new information in the study of innate immunity.

## Supplemental Information

10.7717/peerj.14870/supp-1 Supplemental Information 1 Raw data for qRT-PCR, relative luciferase assay, CFU, survival.

10.7717/peerj.14870/supp-2 Supplemental Information 2 Uncropped Blots for Figs. 1–5.

10.7717/peerj.14870/supp-3 Supplemental Information 3 Att-Luc luciferase reporter system activation. (A) The Flag-IMD plasmid was transfected with the reporter plasmid into S2 cells according to the above concentration gradient. 36 h later, the cells were lysed for Luciferase detection. (B) Flag-IMD was transfected with reporter plasmid in S2 cells at different time points, the cells were lysed for Luciferase detection. Error bars represent SD (*n* = 3). The two-tailed Student’s t test was used to analyze statistical significance. **p < 0.01, ***p < 0.001.

10.7717/peerj.14870/supp-4 Supplemental Information 4 Drs-Luc luciferase reporter system activation. (A) The Flag-TollΔLRR plasmid was transfected with the reporter plasmid into S2 cells according to the above concentration gradient. 36 h later, the cells were lysed for Luciferase detection. (B) Flag-TollΔLRR was transfected with reporter plasmid in S2 cells at different time points, the cells were lysed for Luciferase detection. Error bars represent SD (*n* = 3). The two-tailed Student’s t test was used to analyze statistical significance. **p < 0.01, ***p < 0.001.

10.7717/peerj.14870/supp-5 Supplemental Information 5 Purification of His-IMD and GST-CG4968 proteins. (A,B) Coomassie brilliant blue staining of purified His-IMD and GST-CG4968. 1 μg indicated protein was loaded for each sample.

10.7717/peerj.14870/supp-6 Supplemental Information 6 Identification of CG4968 overexpression and CG4968 knockout transgenic flies. To evaluate the expression levels of CG4968 mRNA, flies bearing overexpression or knockdown of CG4968 exclusively in fat bodies by the ppl-gal4 driver were lysed. Error bars represent SD (*n* = 3). The two-tailed Student’s t test was used to analyze statistical significance. **p < 0.01.

10.7717/peerj.14870/supp-7 Supplemental Information 7 Primers for dsRNA.

10.7717/peerj.14870/supp-8 Supplemental Information 8 Primers for qRT-PCR.

## Additional Information and Declarations

### Data Availability

The following information was supplied regarding data availability: The data are available as Supplemental Files.
