# Supplementary figures and images for: CG4968 positively regulates the immune deficiency pathway by targeting Imd protein in Drosophila
Source: PeerJ. 2023 Feb 7;11:e14870. doi: 10.7717/peerj.14870 (PMC9912943; doi:10.7717/peerj.14870)

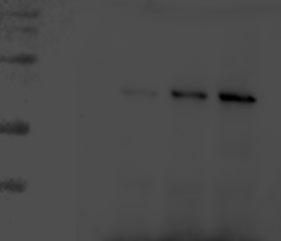

Supplement: Supplemental Information 2 [file peerj-11-14870-s002.zip › Figure 1/FIgure 1C Tubulin.PNG]

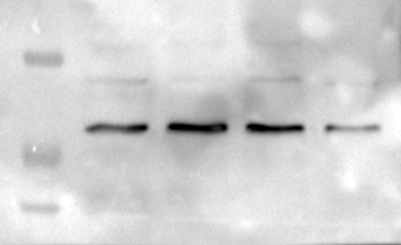

Supplement: Supplemental Information 2 [file peerj-11-14870-s002.zip › Figure 1/Figure 1F Flag.PNG]

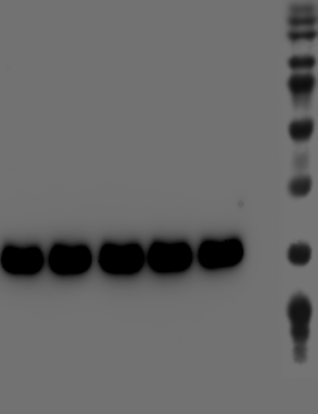

Supplement: Supplemental Information 2 [file peerj-11-14870-s002.zip › Figure 1/Figure 1F Tubulin.tif]

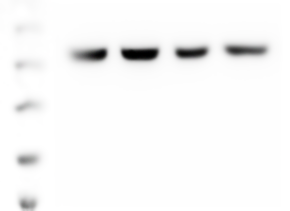

Supplement: Supplemental Information 2 [file peerj-11-14870-s002.zip › Figure 1/Figure1C Tubulin.PNG]

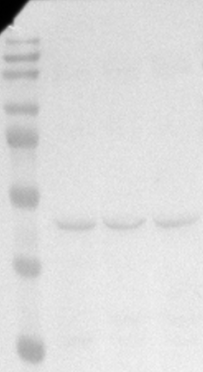

Supplement: Supplemental Information 2 [file peerj-11-14870-s002.zip › Figure 1/Figure1c Flag.PNG]

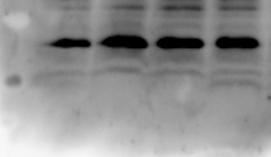

Supplement: Supplemental Information 2 [file peerj-11-14870-s002.zip › Figure 2/Figure 2A Falg.png]

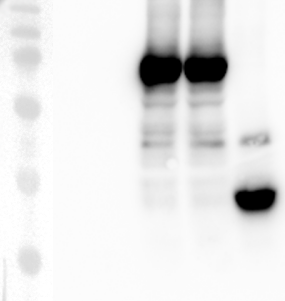

Supplement: Supplemental Information 2 [file peerj-11-14870-s002.zip › Figure 2/Figure 2A Myc.PNG]

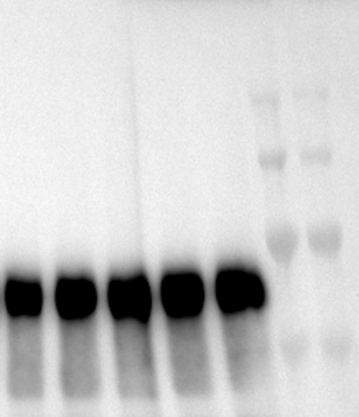

Supplement: Supplemental Information 2 [file peerj-11-14870-s002.zip › Figure 2/Figure 2A Tubulin.PNG]

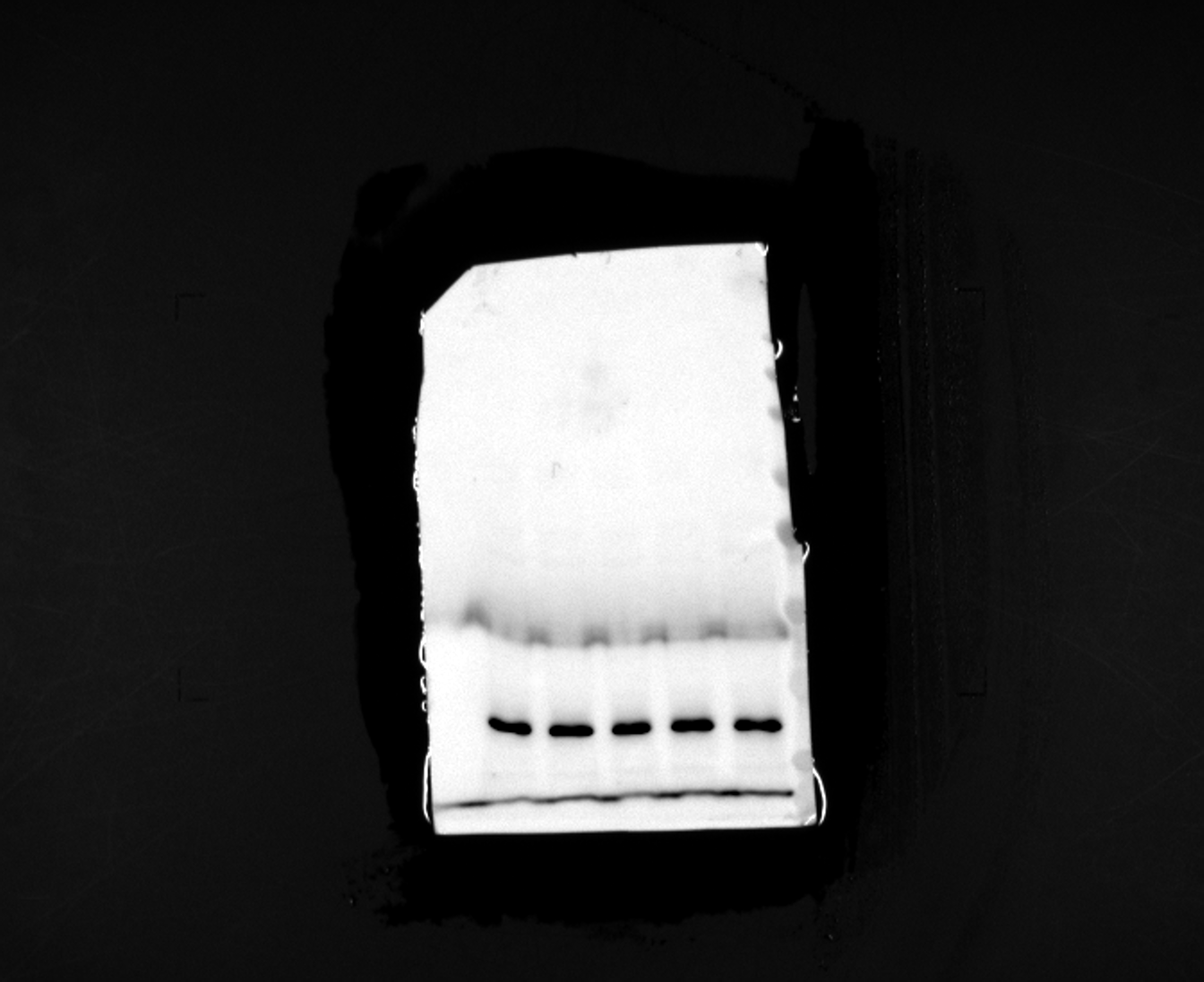

Supplement: Supplemental Information 2 [file peerj-11-14870-s002.zip › Figure 2/Figure 2D Flag.Tif]

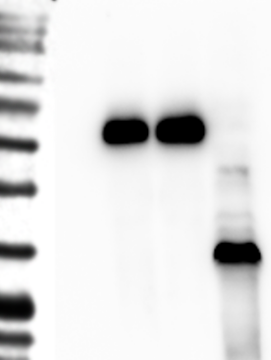

Supplement: Supplemental Information 2 [file peerj-11-14870-s002.zip › Figure 2/Figure 2D Myc.tif]

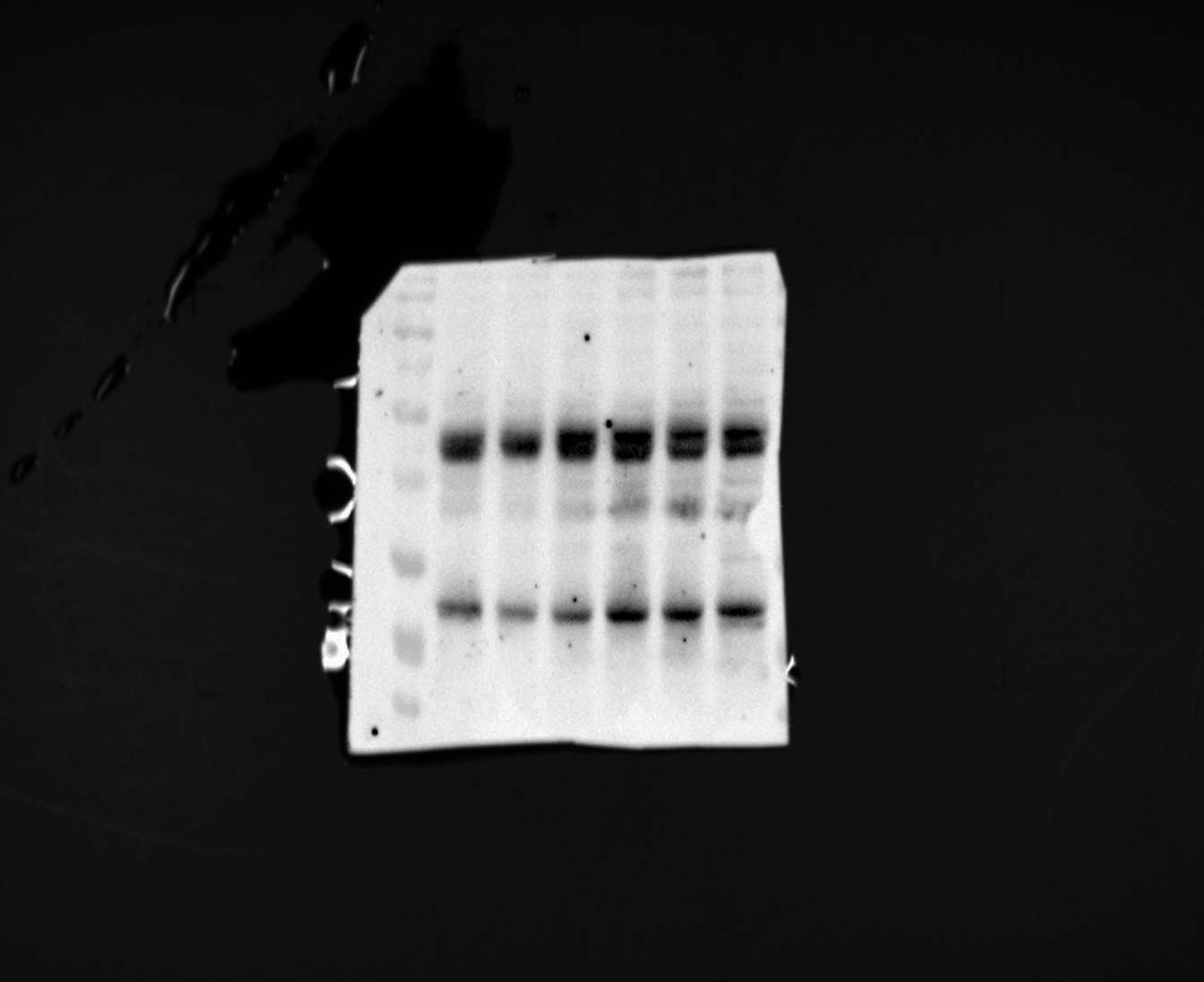

Supplement: Supplemental Information 2 [file peerj-11-14870-s002.zip › Figure 2/Figure 2D Tubulin.Tif]

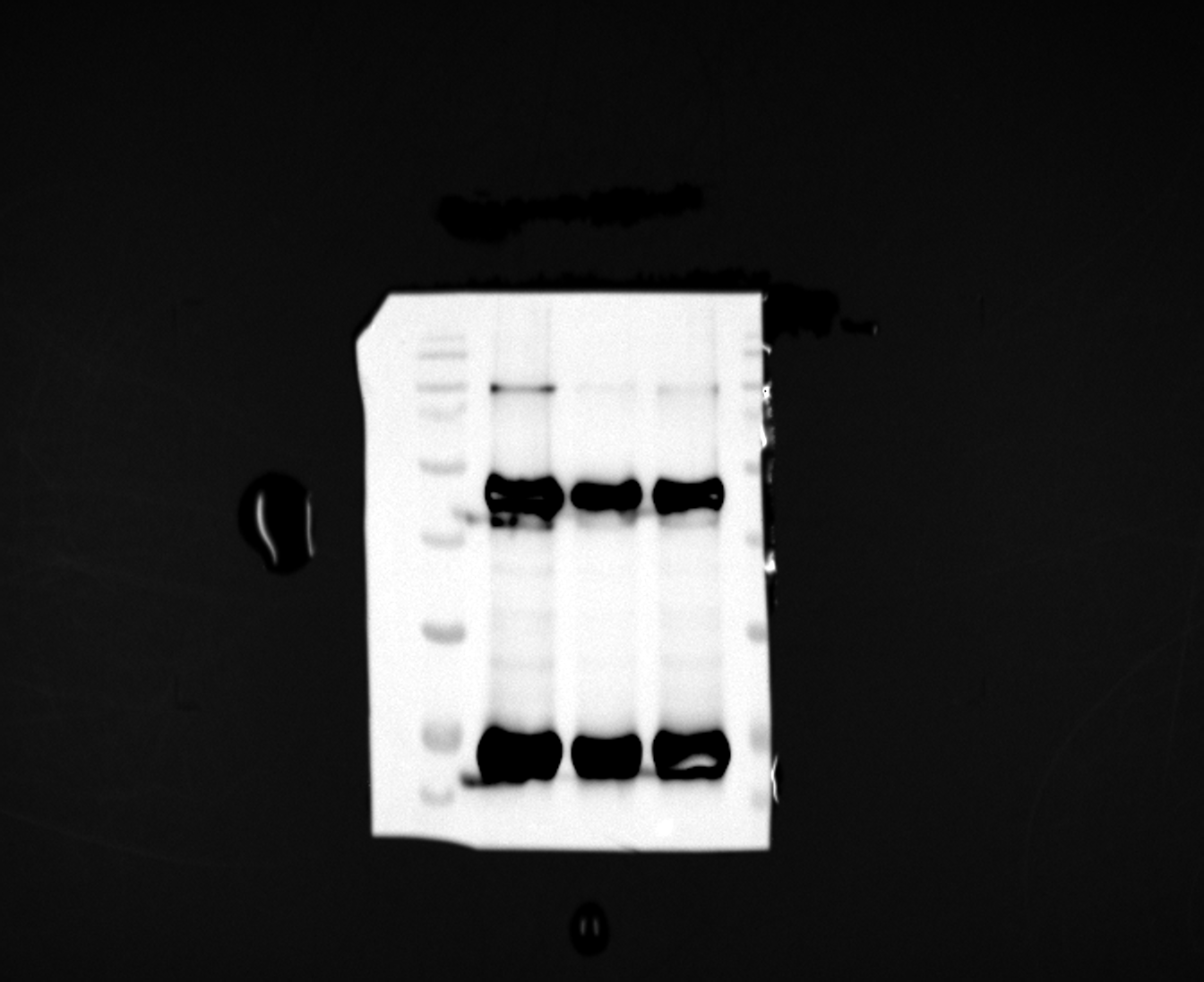

Supplement: Supplemental Information 2 [file peerj-11-14870-s002.zip › Figure 3/Figure 3A Flag.Tif]

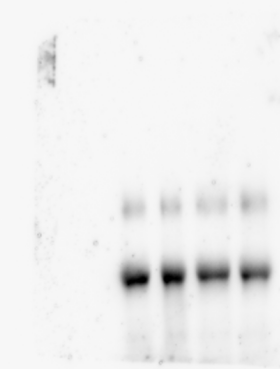

Supplement: Supplemental Information 2 [file peerj-11-14870-s002.zip › Figure 3/Figure 3A Tubulin.PNG]

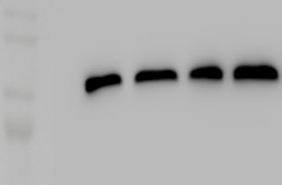

Supplement: Supplemental Information 2 [file peerj-11-14870-s002.zip › Figure 3/Figure 3D Flag.tif]

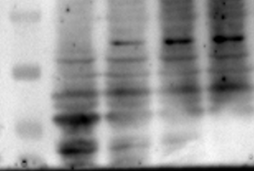

Supplement: Supplemental Information 2 [file peerj-11-14870-s002.zip › Figure 3/Figure 3D Myc.PNG]

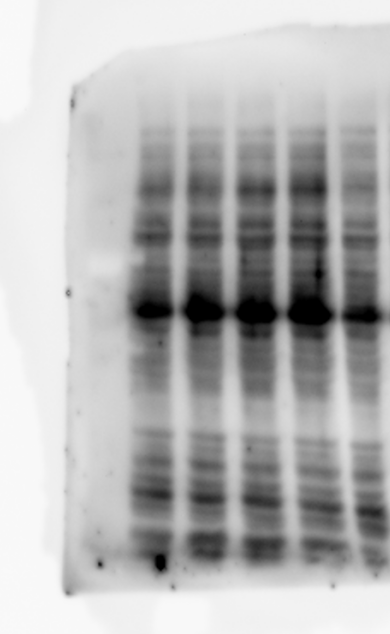

Supplement: Supplemental Information 2 [file peerj-11-14870-s002.zip › Figure 3/Figure 3D Tubulin.PNG]

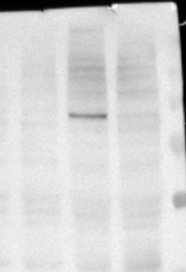

Supplement: Supplemental Information 2 [file peerj-11-14870-s002.zip › Figure 4/FIgure 4A IP Myc.PNG]

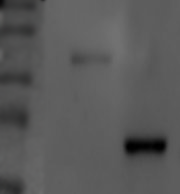

Supplement: Supplemental Information 2 [file peerj-11-14870-s002.zip › Figure 4/Figure 4A IP-Flag.png]

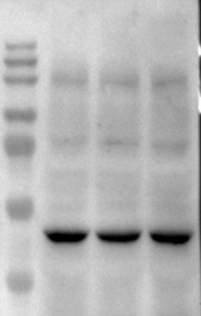

Supplement: Supplemental Information 2 [file peerj-11-14870-s002.zip › Figure 4/Figure 4A Lystae Flag.PNG]

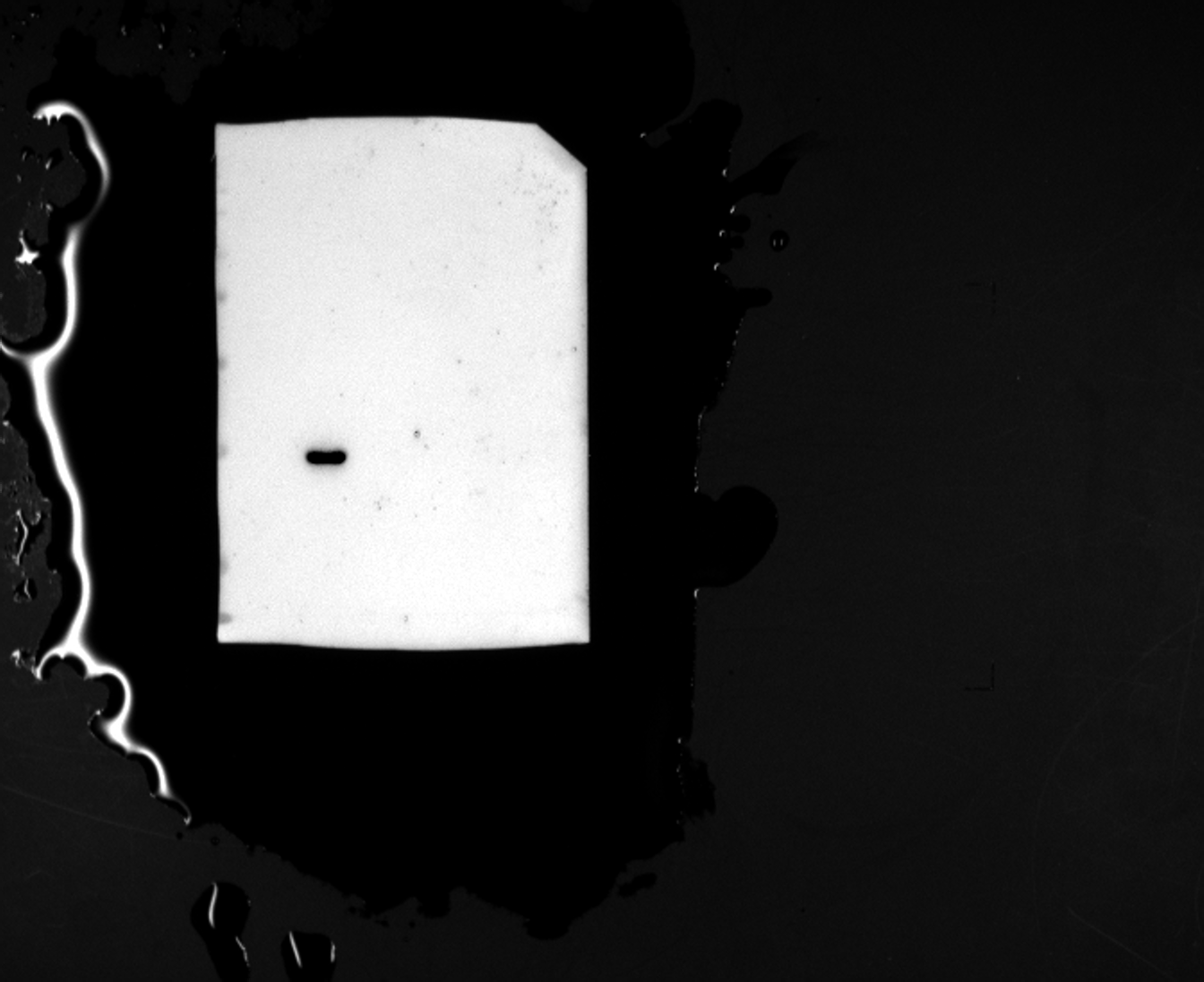

Supplement: Supplemental Information 2 [file peerj-11-14870-s002.zip › Figure 4/Figure 4B His.Tif]

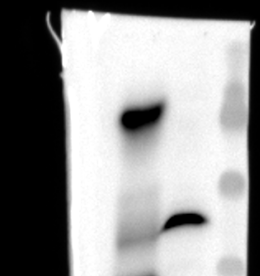

Supplement: Supplemental Information 2 [file peerj-11-14870-s002.zip › Figure 4/Figure 4B GST.PNG]

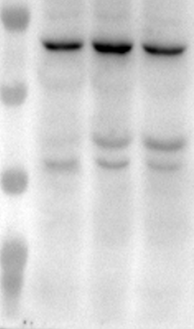

Supplement: Supplemental Information 2 [file peerj-11-14870-s002.zip › Figure 5/FIgure 5B IP Flag.PNG]

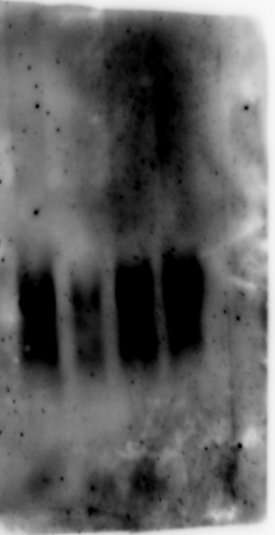

Supplement: Supplemental Information 2 [file peerj-11-14870-s002.zip › Figure 5/Figure 5A IP HA.PNG]

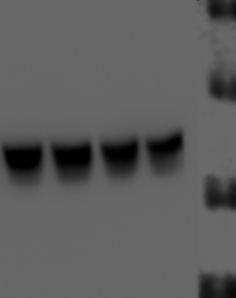

Supplement: Supplemental Information 2 [file peerj-11-14870-s002.zip › Figure 5/Figure 5A Lysate Flag.PNG]

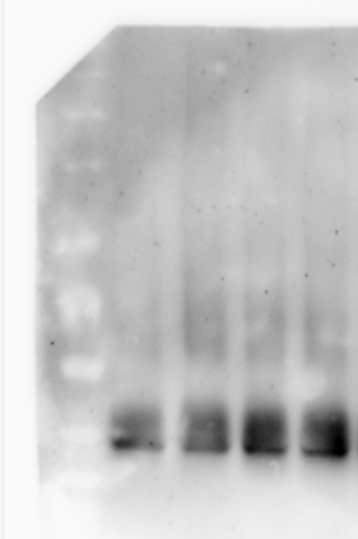

Supplement: Supplemental Information 2 [file peerj-11-14870-s002.zip › Figure 5/Figure 5A Lysate HA.PNG]

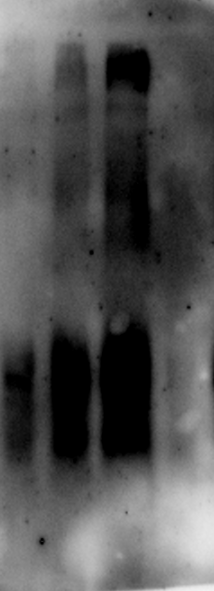

Supplement: Supplemental Information 2 [file peerj-11-14870-s002.zip › Figure 5/Figure 5B IP HA.PNG]

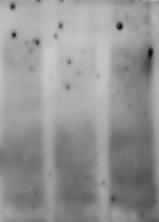

Supplement: Supplemental Information 2 [file peerj-11-14870-s002.zip › Figure 5/Figure 5B lysate-HA.png]

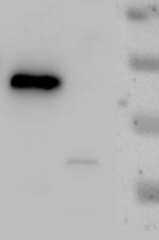

Supplement: Supplemental Information 2 [file peerj-11-14870-s002.zip › Figure 5/Figure 5B lysate-Myc.png]

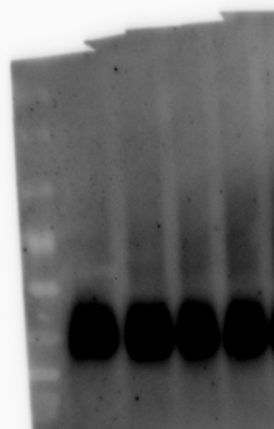

Supplement: Supplemental Information 2 [file peerj-11-14870-s002.zip › Figure 5/Figure 5C IP HA.PNG]

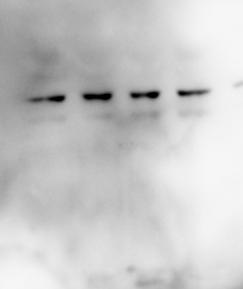

Supplement: Supplemental Information 2 [file peerj-11-14870-s002.zip › Figure 5/Figure 5C IP-Flag.png]

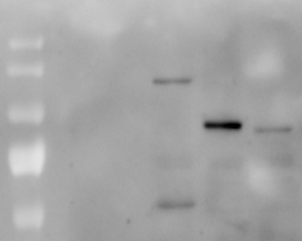

Supplement: Supplemental Information 2 [file peerj-11-14870-s002.zip › Figure 5/Figure 5C Lysate Myc.PNG]

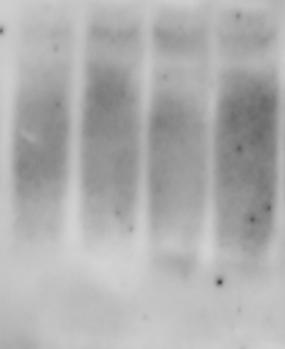

Supplement: Supplemental Information 2 [file peerj-11-14870-s002.zip › Figure 5/Figure 5C lysate-HA.png]

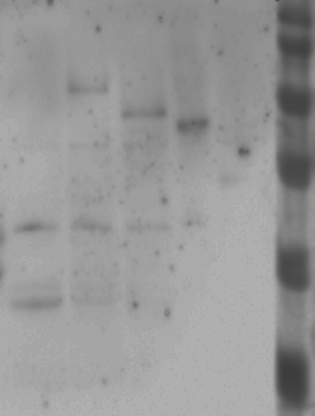

Supplement: Supplemental Information 2 [file peerj-11-14870-s002.zip › Figure 5/Flag 5A Lysate Myc.PNG]

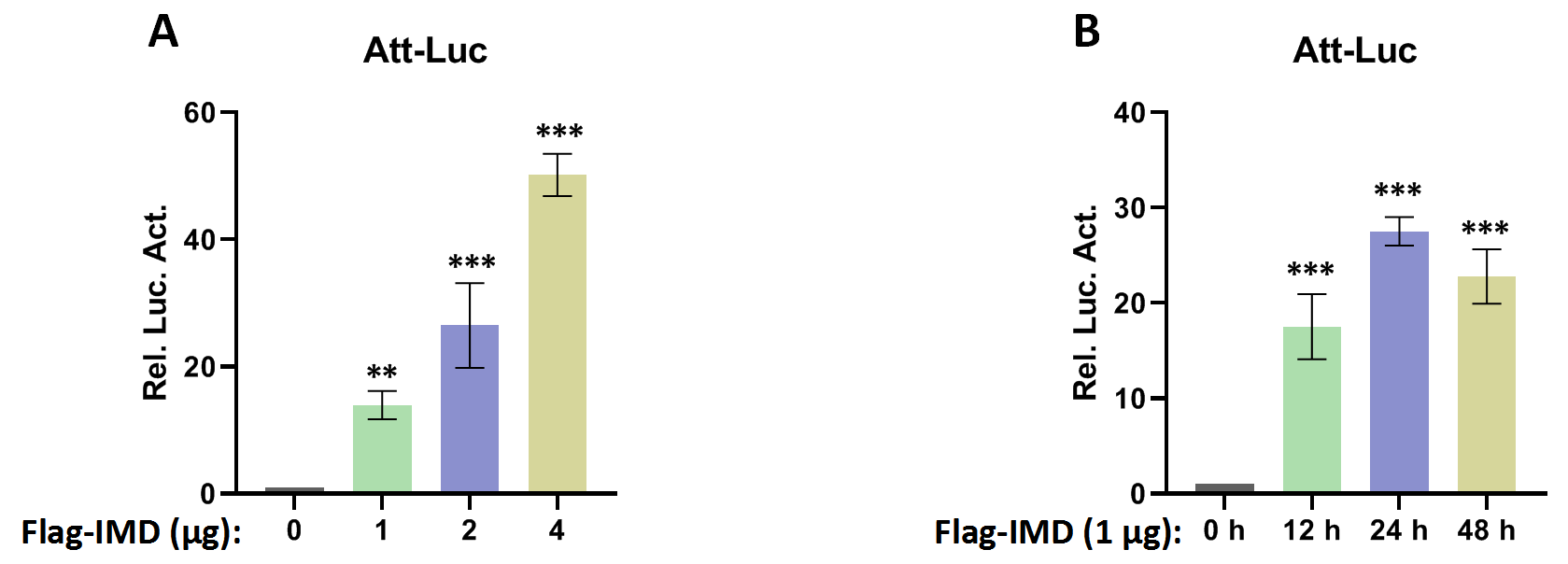

Supplement: Supplemental Information 3 — (A) The Flag-IMD plasmid was transfected with the reporter plasmid into S2 cells according to the above concentration gradient. 36 h later, the cells were lysed for Luciferase detection. (B) Flag-IMD was transfected with reporter plasmid in S2 cells at different time points, the cells were lysed for Luciferase detection. Error bars represent SD (n = 3). The two-tailed Student’s t test was used to analyze statistical significance. **p < 0.01, ***p < 0.001. [file peerj-11-14870-s003.png]

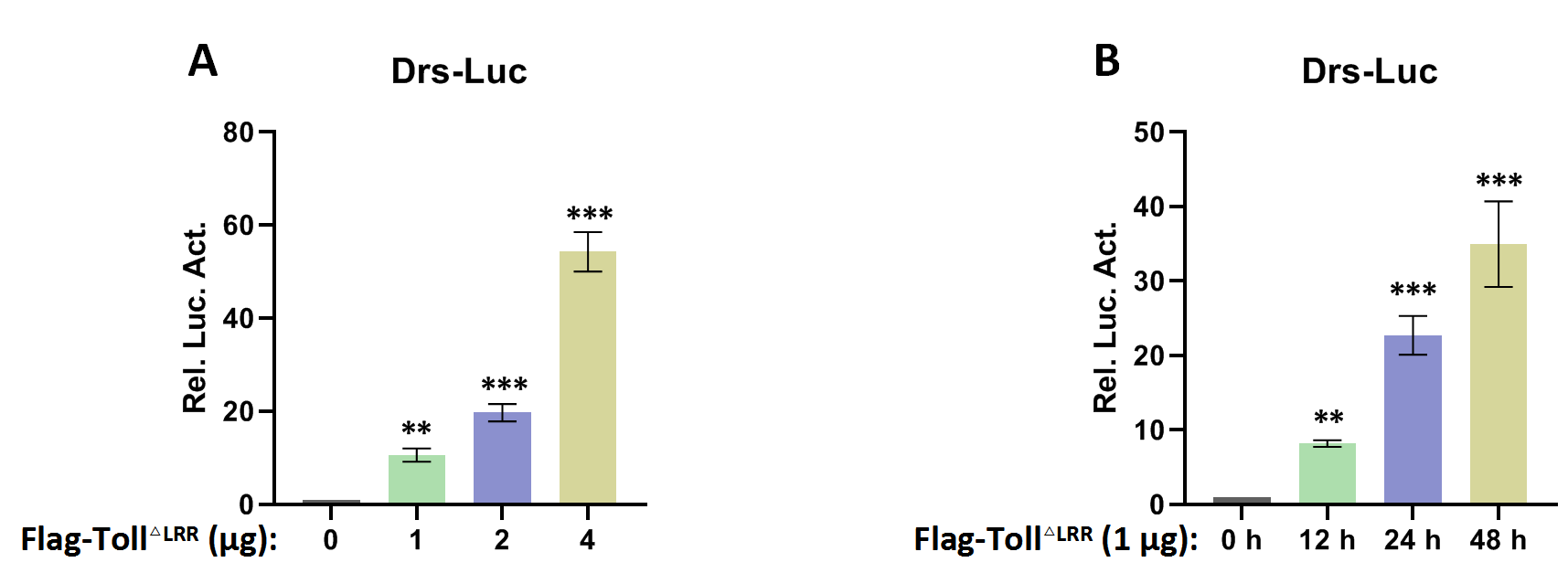

Supplement: Supplemental Information 4 — (A) The Flag-TollΔLRR plasmid was transfected with the reporter plasmid into S2 cells according to the above concentration gradient. 36 h later, the cells were lysed for Luciferase detection. (B) Flag-TollΔLRR was transfected with reporter plasmid in S2 cells at different time points, the cells were lysed for Luciferase detection. Error bars represent SD (n = 3). The two-tailed Student’s t test was used to analyze statistical significance. **p < 0.01, ***p < 0.001. [file peerj-11-14870-s004.png]

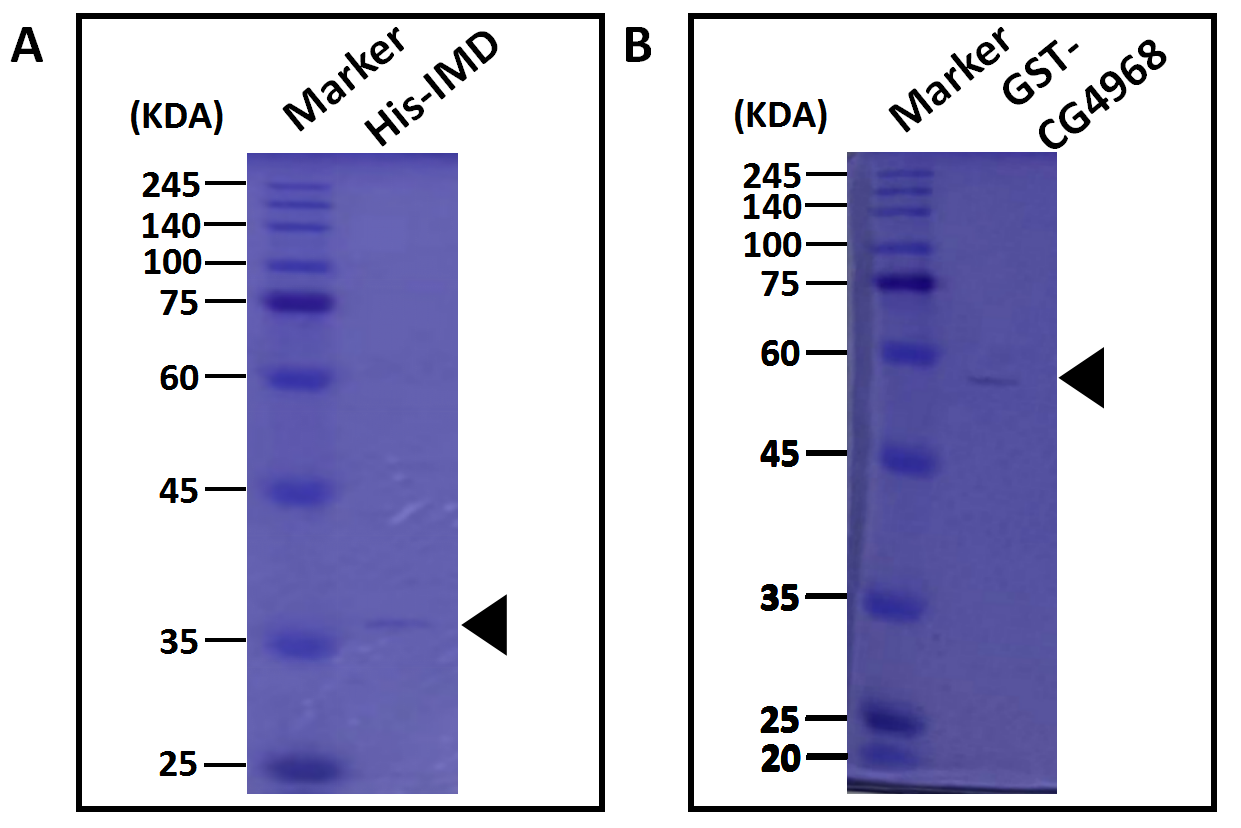

Supplement: Supplemental Information 5 — (A,B) Coomassie brilliant blue staining of purified His-IMD and GST-CG4968. 1 μg indicated protein was loaded for each sample. [file peerj-11-14870-s005.png]

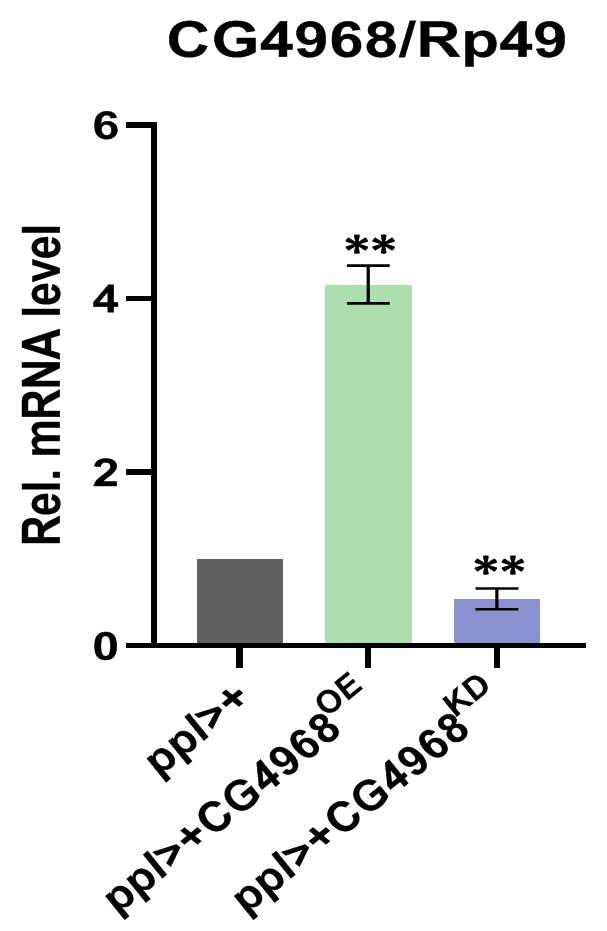

Supplement: Supplemental Information 6 — To evaluate the expression levels of CG4968 mRNA, flies bearing overexpression or knockdown of CG4968 exclusively in fat bodies by the ppl-gal4 driver were lysed. Error bars represent SD (n = 3). The two-tailed Student’s t test was used to analyze statistical significance. **p < 0.01. [file peerj-11-14870-s006.png]
