# Supplementary material for: CG4968 positively regulates the immune deficiency pathway by targeting Imd protein in Drosophila
Source: PeerJ. 2023 Feb 7;11:e14870. doi: 10.7717/peerj.14870 (PMC9912943; doi:10.7717/peerj.14870)
Supplement: Supplemental Information 7 [file peerj-11-14870-s007.docx]

| **Genes** | **forward** **5'-3'** | **reverse** **5'-3'** |
| --- | --- | --- |
| GFP | CTCACTATAGGGAGAAGCAAGGGC  GAGGAGCTGTT | CTCACTATAGGGAGAGGTAGTGGTTG  TCGGGCAG |
| CG4968- 1 | ACTATAGGGAGAATGGAGCCGTTT | ACTATAGGGAGATTTTTTGAACTC |
| CG4968-3’UTR | ACTATAGGGAGAATTGCTCTCAGT | ACTATAGGGAGAGCAAATAAATGG |
