# Supplementary material for: CG4968 positively regulates the immune deficiency pathway by targeting Imd protein in Drosophila
Source: PeerJ. 2023 Feb 7;11:e14870. doi: 10.7717/peerj.14870 (PMC9912943; doi:10.7717/peerj.14870)
Supplement: Supplemental Information 8 [file peerj-11-14870-s008.docx]

| **Genes** | **forward** **5'-3'** | **reverse** **5'-3'** |
| --- | --- | --- |
| Attacin | TGGTCATGGTGCCTCTTTG | GATTGTGTCTGCCATTGTTGA |
| Cecropin | CTTCGTTTTCGTCGCTCTC | TTTTCTTGCCAATTTTCTTCAG |
| Drosomycin | AGTACTTGTTCGCCCTCTTCG | GGTCTCGTTGTCCCAGACG |
| Metchnikowin | CAGTGCTGGCAGAGCCTCAT | ATAAATTGGACCCGGTCTTG |
| Rp49 | CTTCATCCGCCACCAGTC | GGCGACGCACTCTGTTGT |
| CG4968 | ATGGCAATCGCGACGAACTA | CTCAATGGTGAGGTCGCCTT |
